# Genomic analysis of oral *Campylobacter concisus* strains identified a potential bacterial molecular marker associated with active Crohn’s disease

**DOI:** 10.1038/s41426-018-0065-6

**Published:** 2018-04-11

**Authors:** Fang Liu, Rena Ma, Chin Yen Alfred Tay, Sophie Octavia, Ruiting Lan, Heung Kit Leslie Chung, Stephen M. Riordan, Michael C. Grimm, Rupert W. Leong, Mark M. Tanaka, Susan Connor, Li Zhang

**Affiliations:** 1 School of Biotechnology and Biomolecular Sciences, University of New South Wales, Sydney, NSW Australia; 2 Helicobacter Research Laboratory, Marshall Centre for Infectious Diseases Research and Training, School of Pathology and Laboratory Medicine, University of Western Australia, Perth, WA Australia; 3 Gastrointestinal and Liver Unit, Prince of Wales Hospital, University of New South Wales, Sydney, NSW Australia; 4 St George and Sutherland Clinical School, University of New South Wales, Sydney, NSW Australia; 5 Concord Hospital, University of New South Wales, Sydney, NSW Australia; 6 Liverpool Hospital, University of New South Wales, Sydney, NSW Australia

## Abstract

*Campylobacter concisus* is an oral bacterium that is associated with inflammatory bowel disease (IBD) including Crohn’s disease (CD) and ulcerative colitis (UC). *C. concisus* consists of two genomospecies (GS) and diverse strains. This study aimed to identify molecular markers to differentiate commensal and IBD-associated *C. concisus* strains. The genomes of 63 oral *C. concisus* strains isolated from patients with IBD and healthy controls were examined, of which 38 genomes were sequenced in this study. We identified a novel secreted enterotoxin B homologue, Csep1. The *csep1* gene was found in 56% of GS2 *C. concisus* strains, presented in the plasmid pICON or the chromosome. A six-nucleotide insertion at the position 654–659 bp in *csep1* (*csep1*-*6bpi*) was found. The presence of *csep1*-*6bpi* in oral *C. concisus* strains isolated from patients with active CD (47%, 7/15) was significantly higher than that in strains from healthy controls (0/29, *P* = 0.0002), and the prevalence of *csep1*-*6bpi* positive *C. concisus* strains was significantly higher in patients with active CD (67%, 4/6) as compared to healthy controls (0/23, *P* = 0.0006). Proteomics analysis detected the Csep1 protein. A *csep1* gene hot spot in the chromosome of different *C. concisus* strains was found. The pICON plasmid was only found in GS2 strains isolated from the two relapsed CD patients with small bowel complications. This study reports a *C. concisus* molecular marker (*csep1*-*6bpi*) that is associated with active CD.

## Introduction

*Campylobacter concisus* is a Gram-negative bacterium that is associated with inflammatory bowel disease (IBD), due to its significantly higher prevalence in the intestinal tissues of patients with IBD^1–4^. IBD is a chronic inflammatory condition of the gastrointestinal tract with Crohn’s disease (CD) and ulcerative colitis (UC) being the two major clinical forms^5^. In addition to IBD, *C. concisus* may also have a role in diarrhoeal disease as this bacterium was frequently isolated from the diarrhoeal stool samples^6–9^.

*C. concisus* is an oral bacterium, which is present in the oral cavity of nearly every individual including both patients with IBD and healthy controls^10^. Some individuals are colonised by multiple *C. concisus* strains in the oral cavity, which are more often seen in patients with active IBD^11^. There are no distinct oral or enteric *C. concisus* strain clusters and *C. concisus* strains in the intestinal tissues of patients with IBD were found to originate from oral *C. concisus* strains^12^. Some oral *C. concisus* strains were able to invade intestinal epithelial cells and induce epithelial production of IL-8, suggesting that translocation of these oral virulent *C. concisus* strains from the oral cavity into the intestinal tract may cause intestinal inflammation^12–15^.

*C. concisus* consists of two genomospecies (GS), which can be consistently divided based on the analysis of 23S rRNA gene, housekeeping genes and the core genome^7,16–22^. Both GS1 and GS2 contain diverse *C. concisus* strains^23^. Two *C. concisus* virulence factors have been characterised. Phospholipase A was shown to damage the membrane of mammalian cells; and prophage-encoded zonula occludens toxin (Zot) was found to cause prolonged damage to the intestinal epithelial barrier and enhance the responses of macrophages to other enteric bacterial species^24–26^. However, the prevalence of these virulence factors was not associated with IBD^11^. Currently, there are no available bacterial molecular markers that can differentiate commensal *C. concisus* strains from those that are associated with IBD; such markers were investigated in this study. Through genomic analysis, we identified a novel molecular marker in oral *C. concisus* strains that is associated with active CD.

## Results

### The genomes of 38 *C. concisus* strains sequenced in this study

The genomes of 63 oral *C. concisus* strains isolated from saliva samples of 19 patients with IBD (6 active CD, 6 active UC and 7 CD patients in remission) and 23 healthy controls were examined in this study (Table 1). Of the 63 genomes of oral *C. concisus* strains, 38 genomes were sequenced in this study and the remaining 25 genomes (5 GS1 and 20 GS2 strains) were obtained from National Center for Biotechnology Information (NCBI) database^23^.

**Table 1:** *C. concisus* strains used in this study

Strain id	Health status	Disease state	Age/sex	Montreal classification	Current treatment	GS	*Csep1* ^P^	*Csep1* ^C^	N50	Genome size (bp)	No. of contigs	Coverage
P1CDO2	CD	Active	2/M	L2 and L4		2	—	T	80,888	1.99	36	67
P1CDO3						2	—	P^a^	63,559	2.01	53	71
P2CDO3	CD	Relapse, active	19/M	L3 and L4 Previous ileocolonic resection due to stricture		2	—	P^a^				
P2CDO4						2	P^a^	P^a^		2.10	2	42
P2CDO-S6						2	—	P^a^				
P20CDO-S1	CD	Relapse, active	22/M	L1 Previous ileocolonic resection due to stricture		2	—	P				
P20CDO-S2						2	P^a^	—				
P20CDO-S3						2	P^a^	P				
P20CDO-S4						1	—	—				
P21CDO-S1	CD	Relapse, active	21/M	L1 and L4		2	—	—				
P21CDO-S2						2	—	P				
P21CDO-S4						2	—	T				
P10CDO-S1	CD	New case, active	19/M	L3		1	—	—	275,885	1.81	13	312
P10CDO-S2						1	—	—	428,977	1.92	12	298
P11CDO-S1^b^	CD	New case, active	33/M	L3		2	—	P^a^	136,390	2.02	27	295
P3UCO1	UC	New case, active	23/M	Extensive, S1		1	—	—				
P7UCO-S2	UC	New case, active	65/M	Left sided, S2		2	—	P	226,478	1.97	26	327
P13UCO-S1	UC	New case, active	22/M	Extensive, S1		2	—	P	130,673	2.00	34	324
P13UCO-S3						2	—	P				
P15UCO-S2	UC	New case, active	39/M	Extensive, S1		2	—	—				
P16UCO-S1	UC	New case, active	67/M	Extensive, S1		2	—	—	181,794	1.92	24	89
P16UCO-S2						2	—	P	341,483	1.98	11	122
P26UCO-S1	UC	New case, active	34/M	Extensive, S1		2	—	P^a^	122,849	2.08	41	290
P26UCO-S2						1	—	—	125,041	1.87	28	345
P6CDO1	CD	Remission	13/M		Mesalazine	2	—	T	348,922	2.03	21	125
P18CDO-S1	CD	Remission	14/F		Azathioprine	2	—	P	32,428	1.91	94	93
P19CDO-S1	CD	Remission	9/M		Mesalazine, azathioprine and iron supplements	1	—	—	1,174,594	1.81	9	150
P24CDO-S2	CD	Remission	20/F		Azathioprine	2	—	NCS				
P24CDO-S3						2	—	NCS				
P24CDO-S4						2	—	NCS				
P25CDO-S3	CD	Remission	71/F		Azathioprine	1	—	—	273,413	1.95	18	417
P27CDO-S1	CD	Remission	16/F		Azathioprine	2	—	P	205,442	2.00	29	312
P27CDO-S2						1	—	—	201,601	1.83	18	257
P28CDO-S1	CD	Remission	17/M		Cotrimoxazole, tacrolimus, calcium and fish oil	1	—	—	1,034,549	1.96	10	71
H1O1	Healthy		23/F			1	—	—				
H3O1	Healthy		58/M			2	—	P	211,732	1.94	22	191
H7O-S1	Healthy		4/M			2	—	T	346,163	1.94	17	127
H9O-S1	Healthy		27/F			2	—	P	203,101	2.09	19	154
H9O-S2						2	—	P				
H10O-S1	Healthy		18/M			1	—	—	267,337	1.84	13	77
H11O-S1	Healthy		21/F			2	—	T	128,646	1.98	26	81
H11O-S2						2	—	P	200,644	1.95	18	142
H12O-S1	Healthy		16/M			1	—	—	687,684	1.92	11	211
H14O-S1	Healthy		41/F			2	—	—				
H15O-S1	Healthy		60/F			1	—	—	194,730	1.84	14	574
H16O-S1	Healthy		23/F			2	—	—	262,245	1.96	19	123
H17O-S1	Healthy		12/M			1	—	—				
H19O-S1	Healthy		22/F			2	—	NCS	197,598	2.01	31	342
H20O-S1	Healthy		22/F			2	—	P	131,516	2.00	26	174
H21O-S1	Healthy		25/F			2	—	P				
H21O-S2						2	—	—				
H21O-S3						1	—	—				
H21O-S5						2	—	T				
H22O-S1	Healthy		65/M			2	—	NCS				
H23O-S1	Healthy		62/F			2	—	T				
H24O-S1	Healthy		23/F			1	—	—	39,412	1.76	88	150
H25O-S1	Healthy		9/F			1	—	—	180,040	1.91	21	205
H26O-S1	Healthy		16/M			1	—	—	1,025,414	1.84	6	493
H27O-S1	Healthy		5/M			1	—	—	1,209,207	1.88	10	239
H28O-S1	Healthy		67/F			1	—	—	91,334	1.82	30	267
H28O-S2						1	—	—	360,820	1.82	14	334
H29O-S1	Healthy		13/M			2	—	P	64,541	2.00	47	253
H30O-S1	Healthy		16/M			1	—	—	96,450	1.82	31	315

The 63 *C. concisus* strains in Table 1 were examined for the presence of pICON plasmid, *csep1*^P^, *csep1*^C^ and *csep1*^C2^ genes. The details of the draft genomes of 37 *C. concisus* strains and the complete genome of strain P2CDO4 sequenced in this study were listed. The remaining strains have had their draft genomes sequenced in our previous study^23^. Letters P and H in strain ID indicate strains isolated from patients with inflammatory bowel disease and healthy controls, respectively

*Csep1*^P^ pICON plasmid-encoded *csep1* gene, *Csep1*^C^ chromosomally encoded *csep1* gene, *Csep1*^C2^ second copy of chromosomally encoded *csep1* gene, *CD* Crohn’s disease, *UC* ulcerative colitis, *P* present, *T* truncated protein, *NCS* non-coding sequence, — negative for pICON, *csep1*^P^, *csep1*^C^ or *csep1*^C2^

^a^*Csep1*-*6bpi*

^b^The *csep1*^C2^ gene was only found in strain P11CDO-S1

The sizes of the draft genome of the 37 *C. concisus* strains sequenced using MiSeq method ranged between 1.76 and 2.09 Mb and all draft genomes had more than 50 folds coverage (range 67 to 574). The complete genome of strain P2CDO4, which was sequenced using PacBio method, had a genome coverage of 42 with the genome size being 2.10 Mbp. The details of the *C. concisus* genomes sequenced in this study are summarised in Table 1.

### The genomospecies of the 63 oral *C. concisus* strains

**Fig. 1:**
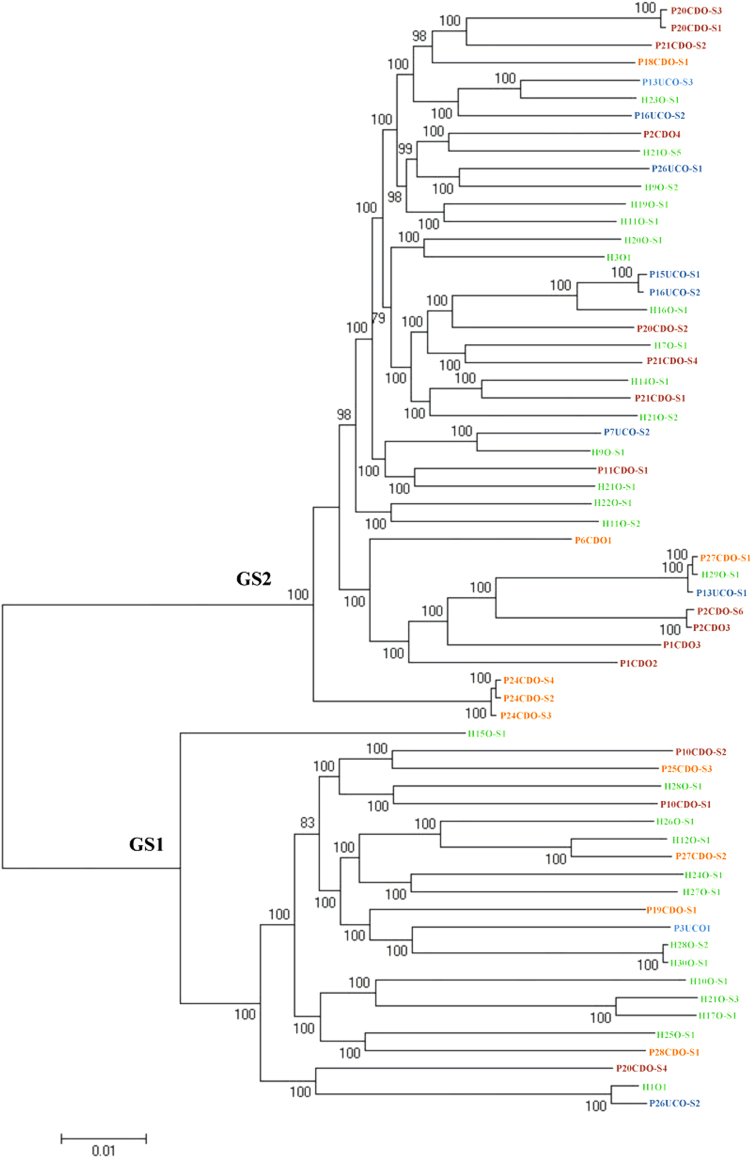
The phylogenetic tree generated based on *C. concisus* core genome. The phylogenetic tree based on the core genome of 63 oral *C. concisus* strains (the genomes of 38 strains were sequenced in this study) was generated to show the GS1 and GS2 strains. The phylogenetic tree was generated using Roary. Strains from active CD, active UC, remission CD and healthy controls were coloured in red, blue, orange and green, respectively. Bootstrap values were generated from 1000 replicates. Bootstrap values of more than 70 were indicated. GS genomospecies

The 63 oral *C. concisus* strains examined in this study were consistently divided into GS1 and GS2 based on the core genome (Fig. 1) and the 23S rRNA gene (Supplementary Figure S1), of which 22 strains belonged to GS1 and 41 strains belonged to GS2 (Fig. 1). The core genome of these 63 strains contained 589 genes that contributed to 29% (589/2077) of the genes present in *C. concisus* strain P2CDO4. The core genomes of GS1 and GS2 *C. concisus* strains consisted of 1014 and 1109 genes, respectively.

The 63 oral *C. concisus* strains included in this study are individual strains; the sequences of their core genome genes were not identical, confirming that they are individual strains (Fig. 1).

### Identification of a novel plasmid pICON in oral *C. concisus* strains isolated from relapsed CD patients with previous ileocecal resection

**Fig. 2:**
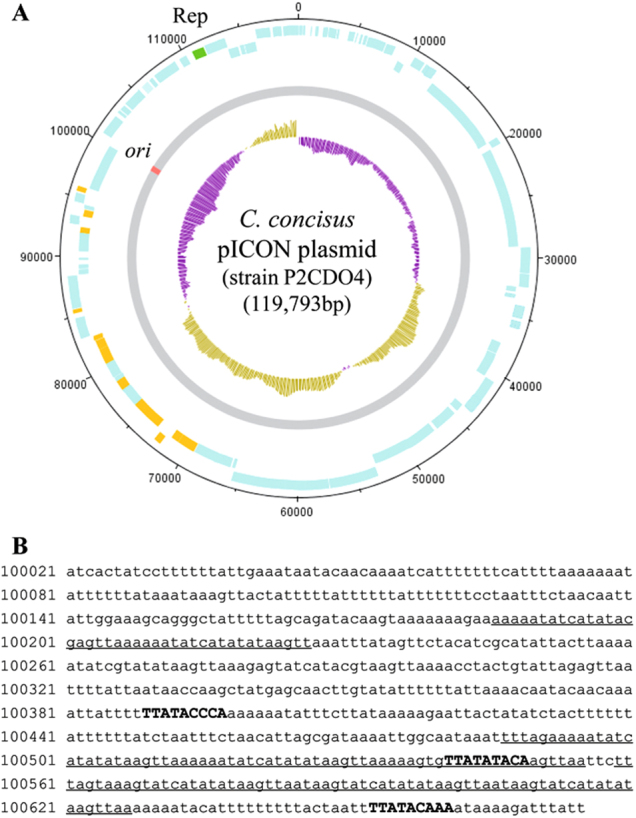
The pICON plasmid. **a** Circularised diagram of the pICON plasmid in *C. concisus* strain P2CDO4. The outer black circle indicated genome base positions around the plasmid, and the outer blue circles represented CDSs on both forward and reverse strands. The plasmid replication initiation protein (Rep) was coloured in green. Predicted secreted proteins were coloured in orange. The grey circle represented the plasmid nucleotide sequence with predicted origin of replication (*ori*) site coloured in red. The inner circle was G+C content plot, G+C content below and above average were coloured in purple and brown, respectively. **b** Position and sequence of the *ori* site. *Ori* was predicted at the position with lowest G+C content. AT-rich regions containing sequence repeats were underlined. *DnaA* boxes were shown in upper case and bold

By comparing the draft genomes of the 63 *C. concisus* strains, we found a highly similar genomic fragment in the draft genomes of strains P2CDO4 (contig 6), P20CDO-S2 (contig 8 and 9) and P20CDO-S3 (contig 9) (Supplementary Figure S2A), which were oral *C. concisus* strains isolated from the two relapsed CD patients with previous ileocecal resection due to small bowel stricture (Table 1). The complete genome of strain P2CDO4 sequenced using the PacBio method confirmed that this fragment was a plasmid (Fig. 2a; Supplementary Figure S2B). The origin of replication (*ori*) site was found at the nucleotide positions between 100,021 and 100,675 bp (655 bp), and contained three *dnaA* boxes including TTATACCCA, TTATATACA and TTATACAAA, and three AT-rich repeats (Fig. 2b). Furthermore, a plasmid-encoded replication initiation protein (CCS77_2118) was found at 110,672–111,694 bp (Fig. 2a). These molecular features were also present in the genomic fragment of strains P20CDO-S2 and P20CDO-S3. Collectively, using previously published criteria for defining a plasmid, these findings confirm that the genomic fragment found in strains P2CDO4, P20CDO-S2 and P20CDO-S3 is a plasmid^27^. We named this plasmid pICON.

Comparison of the nucleotide sequences of the pICON plasmid with the known plasmids in NCBI bacterial genome database did not identify similar plasmids, showing that pICON is a novel plasmid.

Of the 63 oral *C. concisus* strains, only three strains, including P2CDO4, P20CDO-S2 and P20CDO-S3, carried the pICON plasmid, which was consistent in the genome search and PCR detection of pICON plasmid. All the three strains were GS2 *C. concisus* (Table 1). The prevalence of pICON plasmid in patients with active CD was significantly higher than that in healthy controls (2/6 vs. 0/23, *P* = 0.037).

### The Csep1 protein

We compared the proteins encoded by the pICON plasmid in strains P2CDO4, P20CDO-S2 and P20CDO-S3 with known bacterial virulence proteins and found that the protein encoded by gene CCS77_2074 in the pICON plasmid was homologous to *Staphylococcus aureus* enterotoxin B (*E* = 0.04) and predicted to be secreted (Supplementary Table S1 and S2). We named it *C. concisus*-secreted protein 1 (Csep1). We found another Csep1 protein encoded by gene CCS77_0139 in the chromosome of *C. concisus* strain P2CDO4, which had 85% amino acids identical to the Csep1 protein encoded by gene CCS77_2074 in the pICON plasmid. We used Csep1^P^ and Csep1^C^ to differentiate the pICON plasmid-encoded and chromosomally encoded Csep1 proteins.

Except for Csep1^P^, all proteins encoded by the pICON plasmid had an amino acid identity of <40% as compared to proteins encoded by the chromosome of strain P2CDO4, showing that the *csep1* gene in the chromosome was not due to the integration of pICON plasmid into the chromosome.

### The *csep1* gene in different oral *C. concisus* strains and their prevalence in patients with IBD and controls

The *csep1* gene in different *C. concisus* strains was identified by genome search and then confirmed using various PCR methods, which showed consistent results.

The *csep1*^P^ gene was found in the three *C. concisus* strains containing pICON plasmid (P2CDO4, P20CDO-S2 and P20CDO-S3). The *csep1*^C^ gene was found in 22 *C. concisus* strains, all contained one copy of the *csep1*^C^ gene except for strain P11CDO-S1, which contained two copies of the *csep1*^C^ gene (*csep1*^C^ and *csep1*^C2^). Strains P2CDO4 and P20CDO-S3 contained both *csep1*^P^ and *csep1*^C^, strain P20CDO-S2 had *csep1*^P^ but no *csep1*^C^.

**Fig. 3:**
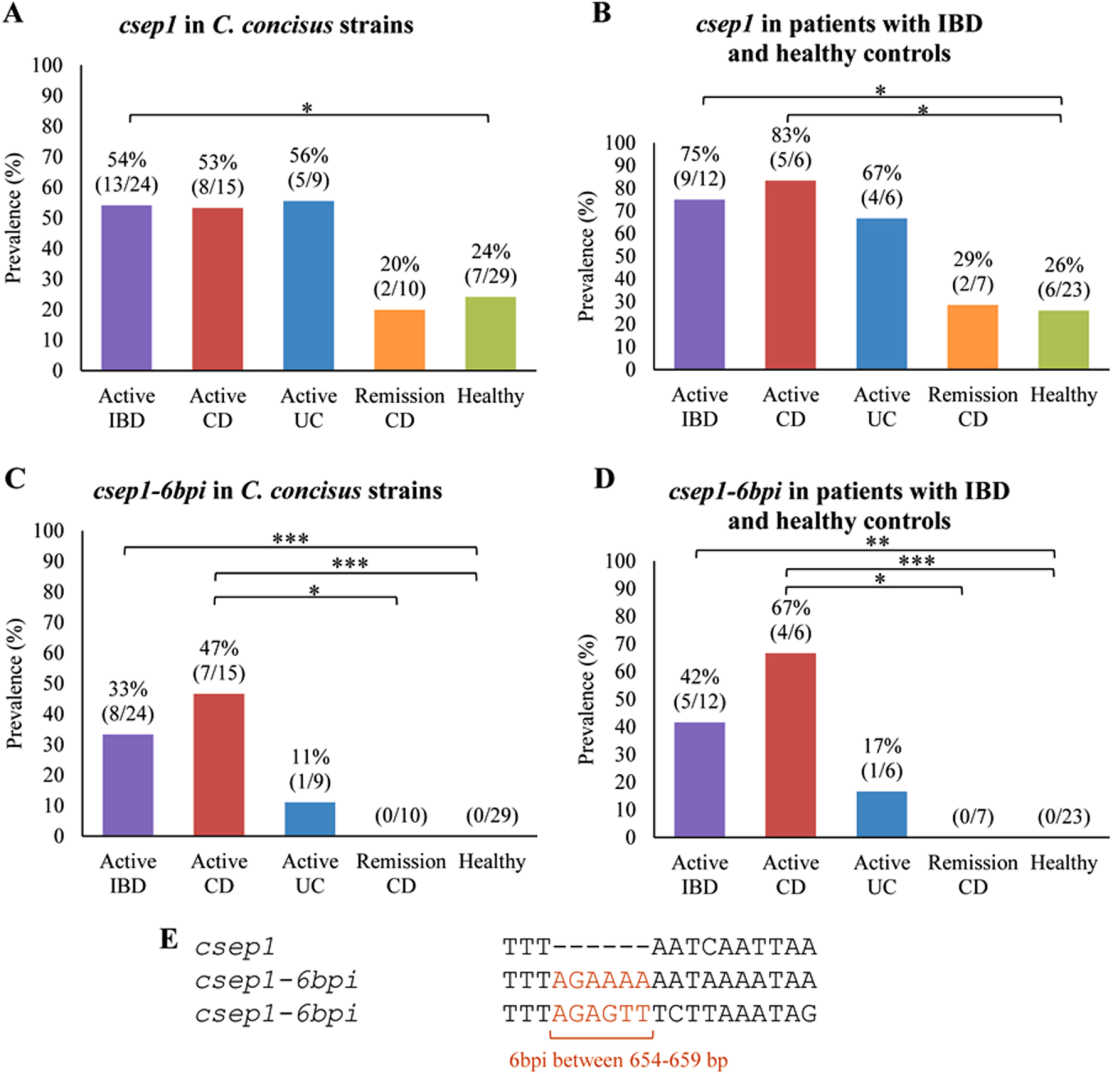
The prevalence of *csep1* and *csep1*-*6bpi* genes in patients with IBD and healthy controls. **a** The prevalence of the *csep1* gene in *C. concisus* strains isolated from active IBD was significantly higher than that from healthy controls (*P* = 0.045). **b** The prevalence of *csep1*-positive *C. concisus* strains from active CD was significantly higher than that from healthy controls (*P* = 0.019). **c** A six bp insertion (6bpi) at position 654–659 bp was mainly found in the *csep1* genes (*csep1*-*6bpi*) from *C. concisus* strains isolated from active CD, not from remission CD and healthy controls. (*P* = 0.02 and *P* = 0.0002, respectively). **d** The prevalence of *csep1*-*6bpi* positive *C. concisus* strains in patients with active CD was significantly higher than that in remission CD and health controls (*P* = 0.021 and *P* = 0.0006, respectively). **e** Majority of the *csep1*-*6bpi* contained AGAAAA between 654 and 659 bp, while only one contained AGAGTT. *Indicates statistical significance (*P < 0.05, **P < 0.01 and ***P < 0.001). CD Crohn’s disease, UC ulcerative colitis

The *csep1* gene (either *csep1*^P^ or *csep1*^C^) was found in GS2 *C. concisus* strains (56%, 23/41) and in none of the GS1 strains. More than half of the oral *C. concisus* strains isolated from patients with IBD (54%, 13/24) contained the *csep1* gene, which was significantly higher than that in the oral *C. concisus* strains isolated from healthy controls (24%, 7/29, *P* = 0.045) (Fig. 3a). The prevalence of *csep1*-positive *C. concisus* strain was significantly higher in patients with active CD (83%, 5/6) as compared to healthy controls (26%, 6/23, *P* = 0.019) (Fig. 3b).

### A six bp insertion in the *csep1* gene is strongly associated with active CD

The sequences of the *csep1* gene in different *C. concisus* strains were compared (Supplementary Figure S3). The *csep1* gene in different *C. concisus* strains had sizes ranging between 651 and 672 bp, encoding proteins of 216–223 amino acids. All Csep1 proteins were predicted to be secreted proteins, containing a signal peptide (Supplementary Figure S4). In addition, 12 strains had truncated *csep1*^C^ or non-coding *csep1*^C^ genes. The truncated *csep1*^C^ genes had stop codons at various positions within the gene and the non-coding *csep1*^C^ genes were gene fragments without a start codon or very short gene fragments. The truncated and non-coding *csep1*^C^ genes and their flanking genes were in the same contig, their presence therefore was not due to assembly. The truncated and non-coding *csep1*^C^ genes were also confirmed by the PCR method targeting the flanking sequences.

Nucleotide insertions were found at six positions in the *csep1* gene in different *C. concisus* strains (Supplementary Figure S3). The six bp insertion at the nucleotide 654–659 bp of the *csep1* gene (*csep1*-*6bpi*) was found in seven oral *C. concisus* strains isolated from patients with active CD, one strain from a patient with active UC and none of the strains from CD patients in remission and healthy controls (Supplementary Figure S3). The presence of *csep1*-*6bpi* gene in oral *C. concisus* strains isolated from patients with active CD (47%, 7/15) was significantly higher than that in oral strains from healthy controls (0/29, *P* = 0.0002) and patients with CD in remission (0/10, *P* = 0.02) (Fig. 3c). The prevalence of *csep1*-*6bpi* positive *C. concisus* strains was significantly higher in patients with active CD (67%, 4/6) as compared to healthy controls (0/23, *P* = 0.0006) and CD patients in remission (0/7, *P* = 0.021) (Fig. 3d). When comparing the prevalence of *csep1*-*6bpi* positive *C. concisus* strain in patients and healthy controls, if an individual is colonised by multiple *csep1*-*6bpi* positive strains, the positivity was counted only once.

Of the eight strains that had the *csep1*-*6bpi*, the six bp insertion sequences were AGAAAA in seven strains and AGAGTT in one strain (Fig. 3e). Both Csep1^P^ and Csep1^C^ from strain P2CDO4 contained AGAAAA.

### Phylogenetic analysis of the *csep1* gene in different oral *C. concisus* strains

**Fig. 4:**
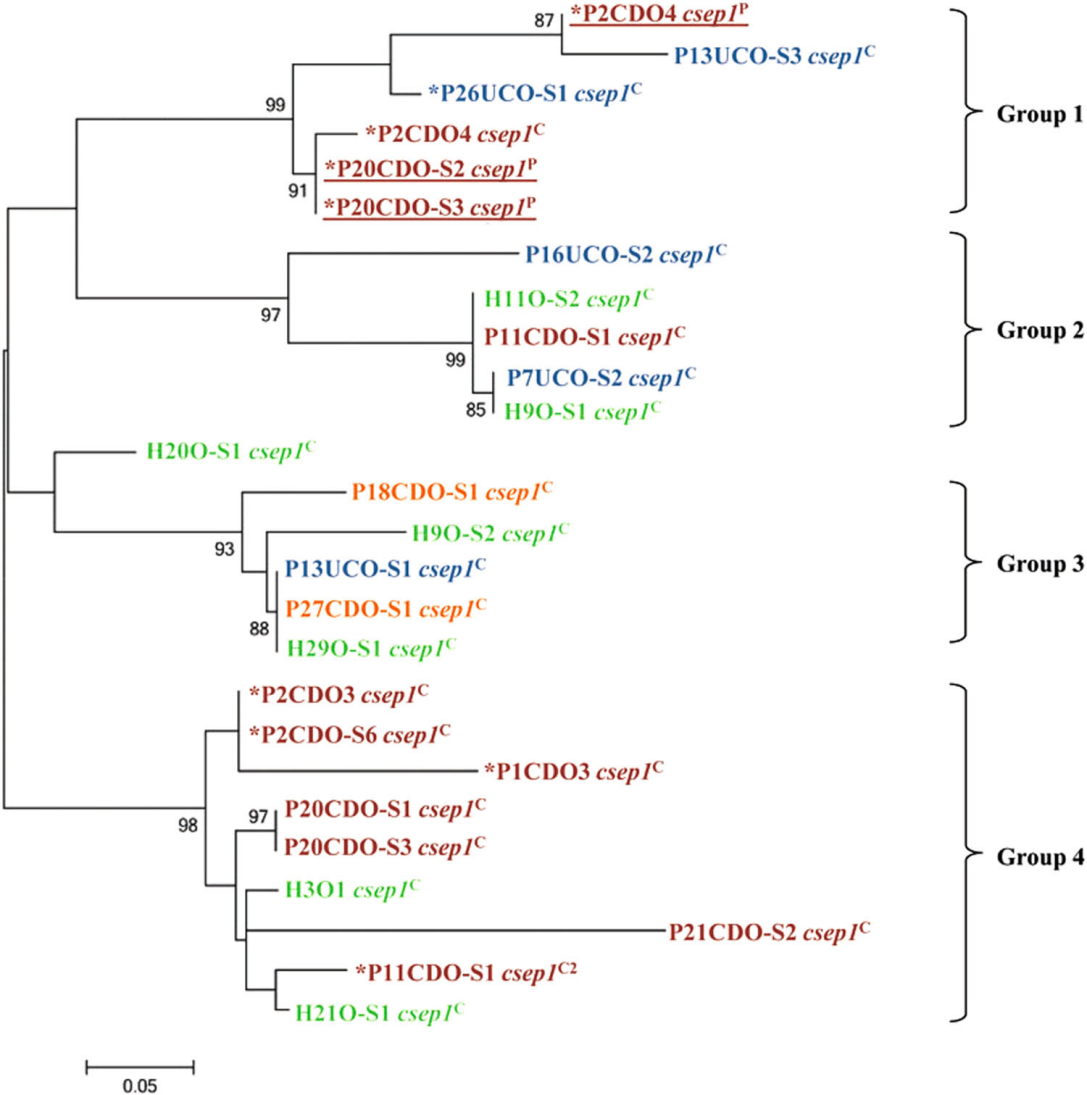
The phylogenetic tree generated based on *csep1* genes in *C. concisus* strains. Maximum likelihood method was used to generate the phylogenetic tree. *Bootstrap* values were generated from 1000 replicates. Bootstrap values of more than 70 were indicated. The *csep1* genes from *C. concisus* strains isolated from patients with active CD, active UC, remission CD and healthy controls were coloured in red, blue, orange and green, respectively. The *csep1*^P^ genes were underlined. *The *csep1*-*6bpi* gene

The phylogenetic tree generated based on the *csep1* gene in different oral *C. concisus* strains formed four groups (groups 1–4, Fig. 4). The *csep1*^P^ and *csep1*^C^ genes did not form distinct groups. Five of the *csep1*-*6bpi* genes (AGAAAA) were in group 1 and the remaining four *csep1*-*6bpi* genes (three AGAAAA and one AGAGTT) were in group 4. The phylogenetic clustering of the *csep1* genes was not consistent with the phylogenetic grouping of the *C. concisus* strains based on their core genomes.

### The *csep1* gene insertion sites in the *C. concisus* genome

To understand the insertion patterns of *csep1*^P^, *csep1*^C^ and *csep1*^C2^ in the *C. concisus* genome, the upstream and downstream flanking genes were compared.

**Fig. 5:**
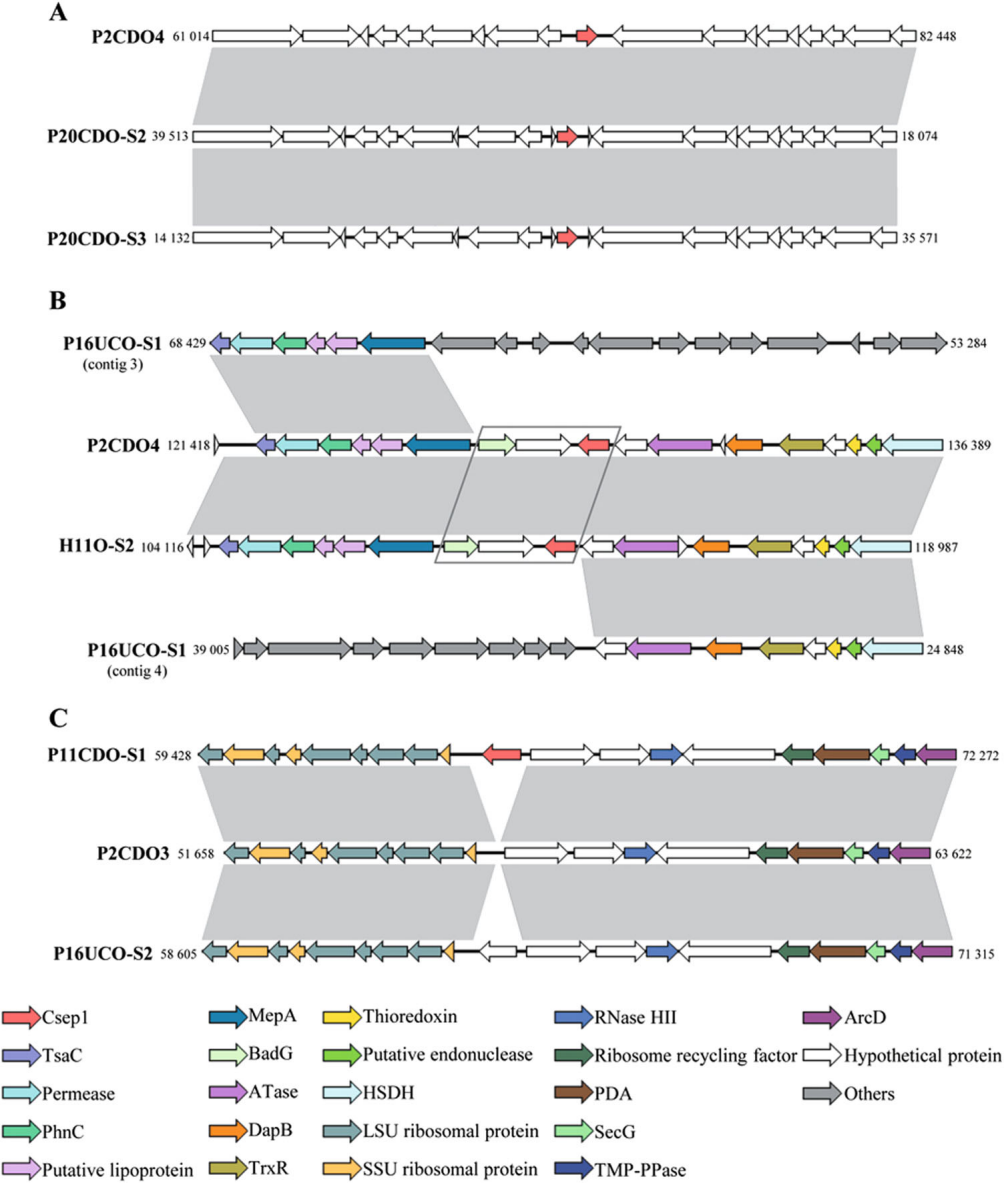
Insertion sites of *csep1* genes in *C. concisus* genomes. **a** The flanking genes of the *csep1*^P^ gene in the pICON plasmid of strain P2CDO4, P20CDO-S2 and P20CDO-S3 were almost identical, indicating the *csep1*^P^ was inserted at the same location in the pICON plasmid of different strains. **b** The flanking genes of the *csep1*^C^ gene in all the *csep1*^C^-positive strains shared similar patterns, strains P2CDO4 and H11O-S2 are shown here as examples. The two genes (boxed) immediately upstream of the *csep1*^C^ gene were absent in all *csep1*^P^-negative strains, while other flanking genes were present, although distantly located, strain P16UCO-S1 is shown here as an example. **c** The *csep1*^C2^ gene was inserted at a rare spot within the chromosome, in which only appeared in strain P11CDO-S1. In the remaining strains with similar flanking gene arrangements, there was either no gene insertion, such as strain P2CDO3; or insertion of other genes such as strain P16UCO-S2. Nucleotide sequences sharing more than 80% identity were shaded in grey. Nucleotide positions referred to the positions within the contig, except strain P2CDO4 which had the genome sequenced without gap. Gene sizes in strains between **a**, **b** and **c** were not on scale. HSDH homoserine dehydrogenase, PDA polysaccharide deacetylase

The *csep1*^P^ was located between 72,094 and 72,762 bp in the pICON plasmid of strain P2CDO4. All three pICON plasmids identified in this study contained the *csep1*^P^ gene and the flanking genes were almost identical (with more than 80% of nucleotide identity), showing that the *csep1*^P^ gene was inserted at the same location in the pICON plasmid in different *C. concisus* strains (Fig. 5a).

The *csep1*^C^ in the chromosome was located between 128,877 and 129,542 bp in strain P2CDO4 (Fig. 5b). Of the 23 copies of the *csep1* gene in the chromosome carried by 22 *C. concisus* strains, 22 copies of the *csep1*^C^ gene were in the same position, demonstrated by their flanking genes which were almost identical (strains P2CDO4 and H11O-S2 were used as examples to show the location of the *csep1*^C^ gene). Most of the flanking genes encode for bacterial enzymes. The flanking genes were also present in the *csep1*^C^-negative strains, but were distantly located (strain P16UCO-S1 was used to show the distantly located flanking genes in Fig. 5b), indicating that gene rearrangement has occurred. Furthermore, the two genes immediately upstream of the *csep1*^C^ gene were absent in all *csep1*^C^ negative strains, these two genes encode for a hypothetical protein and benzoyl-CoA reductase subunit BadG (boxed in Fig. 5b).

P11CDO-S1 is the only strain that carried a second copy of the *csep1* in the chromosome (*csep1*^C2^) and most of the flanking genes encoded ribosomal proteins and bacterial enzymes. Among the remaining 62 *csep1*^C2^-negative strains, 33 strains had other genes inserted at the same position encoding for putative type-IIS restriction/modification enzyme or hypothetical proteins; 26 strains had no insertion; two strains had the flanking genes located distantly; and one strain had contigs ended at the insertion site, thus information regarding gene insertion was unavailable. Strains P11CD-S1, P2CDO3 and P16UCO-S2 were used to show the insertion site of *csep1*^C2^ and the flanking genes (Fig. 5c).

### Detection of Csep1 protein expression in *C. concisus* culture supernatant

Csep1 proteins, including both Csep1^P^ and Csep1^C^, were predicted to be secreted proteins (Supplementary Table S2; Supplementary Figure S4). Using mass spectrometry analysis, both Csep1^P^ and Csep1^C^ were detected in the bacterial culture supernatant of *C. concisus* strain P2CDO4. Unique peptides containing amino acids specific to Csep1^P^ or Csep1^C^ were detected (Csep1^P^: LIEINTRPISTDNAK and NDIDNKTIK; Csep1^C^: NIPAIDLIK; specific amino acids were underlined), common peptides shared between Csep1^P^ and Csep1^C^ were also detected (MLEYGCNELK and TIPEYCYDKK).

### The prevalence of pICON plasmid and *csep1* in other *C. concisus* strains in the public databases

There are genomes of 125 other *C. concisus* strains available from the public databases including 42 GS1 and 83 GS2 *C. concisus* strains. Most of these strains were enteric strains isolated from the stool samples and intestinal biopsies of patients with diarrhoea, CD, UC or healthy individuals. There were 16 oral strains isolated from 5 patients with UC, 3 patients with CD, 1 patient with gingivitis and 4 healthy individuals^23,28–31^. It was not clear whether patients with IBD had active disease and whether they were receiving IBD treatment at the time of *C. concisus* strain isolation, these isolates are therefore not suitable for analysing the prevalence of *csep1*-*6bpi*, as we had previously shown that the drugs such as azathioprine and mercaptopurine used for IBD treatment could inhibit the growth of *C. concisus*^32^.

We examined the presence *csep1* gene and pICON plasmid in these strains. We found that the five oral strains isolated from four healthy individuals were all negative for the *csep1*-*6bpi* gene and the pICON plasmid. There were only two strains had genomic fragments similar to the pICON plasmid, and these two strains were isolated by Kirk et al.^31^ from a patient with UC. However, the contigs of the oral strain from this patient were really short, and the contigs did not cover the full length of the *csep1* gene (Supplementary Figure S5A). The enteric strain had longer contigs (Supplementary Figure S5B), and this strain contained the *csep1*-*6bpi* gene with the flanking genes similar to *csep1*^P^. Overall, these data suggest that these two strains have the pICON plasmid. Interestingly, these strains are GS1 strains, suggesting that pICON plasmid can be transmitted between GS1 and GS2 *C. concisus* strains. However, the genomes sequenced by Kirk et al. were not complete genomes without gaps; therefore, we cannot carry further analysis of the pICON plasmid in their strains.

## Discussion

In this study, we analysed the genomes of 63 oral *C. concisus* strains isolated from patients with IBD and controls and the genomes of 38 *C. concisus* strains were sequenced in this study. We identified a novel bacterial biomarker that is associated with active CD, and this marker was confirmed by PCR methods.

We identified the *C. concisus* Csep1 protein, which is homologous to enterotoxin B encoded by *S. aureus*. Staphylococcal enterotoxin B has multiple pathogenic effects such as inducing diarrhoea and acting as a human superantigen that non-specifically activates T cells to produce a large amount of proinflammatory cytokines^33^. Further analysis found nucleotide insertions in the *csep1* gene in different *C. concisus* strains and the *csep1*-*6bpi* insertion at the position 654–659 bp was only found in oral *C. concisus* strains isolated from patients with active IBD particularly in CD. The prevalence of *csep1*-*6bpi* positive *C. concisus* strains in patients with active CD was significantly higher than that in the healthy controls (*P* = 0.0006). Future studies are needed to assess the effects of *C. concisus* Csep1 protein encoded by the *csep1*-*6bpi* gene on human gastrointestinal epithelial cells and the mucosal immune system, which will provide information regarding whether this protein has a role in the development or pathogenesis of CD. The *csep1* gene was located in the chromosome (*csep1*^C^) or the pICON plasmid (*csep1*^p^). The *csep1*^C^ in the majority of the *C. concisus* strains were at the position 128,877–129,542 bp (nucleotide position in strain P2CDO4), showing that this is a *csep* hot spot. One strain (P11CDO-S1) had a second copy of the *csep1* gene (*csep1*^C2^), which was identified at the location between 1,819,244 and 1,820,490 bp (nucleotide position in strain P2CDO4). The Csep1 was predicted to be a secreted protein, containing a signal peptide (Supplementary Table S2). Proteomics analysis indeed detected Csep1 proteins encoded by the *csep1* gene in both pICON plasmid and the chromosome from the culture supernatant of *C. concisus* strain P2CDO4.

Phylogenetic analysis of the *csep1* gene from different *C. concisus* strains identified four groups. *Csep1*^P^ and *csep1*^C^ did not form distinct groups, showing that they were from the same ancestor. We also compared the flanking genes of the *csep1* gene in both pICON and the chromosome (Fig. 5). The flanking genes of *csep1*^P^ in the pICON plasmid in different strains were nearly identical, suggesting the *csep1*^P^ was transmitted by the plasmid between the strains. The *csep1*^C^ appeared not stable, in 12 GS2 *C. concisus* strains, truncated or non-coded *csep1*^C^ gene was found, implying that the *csep1*^C^ genes in these *C. concisus* strains have undergone mutations (Table 1).

A novel and rare *C. concisus* plasmid, the pICON plasmid, is reported for the first time in this study. Of 63 oral *C. concisus* strains examined in this study, only 3 GS2 strains isolated from 2 relapsed CD patients contained the pICON plasmid. These 2 patients were not related, and their saliva samples were collected from different hospitals. Interestingly, both patients had previous ileocecal resection due to small bowel restriction within 2 years of their diagnosis of CD, suggesting that CD patients colonised by pICON plasmid-positive GS2 *C. concisus* strains may be more likely to develop complications, which should be further investigated.

*C. concisus* consists of two GS^23^. In comparison with the GS1 strains, GS2 strains are better adapted to the human gastrointestinal tract. More GS2 strains were isolated from the saliva samples of patients with IBD as compared to healthy controls and previous studies showed that GS2 *C. concisus* strains were more invasive to human intestinal epithelial cell lines as compared to GS1 strains^34^. Each *C. concisus* GS contained diverse strains, as shown by the number of genes in the GS core genome. We found that *csep1* were present in the chromosome of 56% of oral GS2 *C. concisus* strains, showing that it is possible to further divide GS2 strains into CD-associated strains and the other strains based on this gene.

The *csep1*-*6bpi*-positive *C. concisus* strains were not detected in the seven CD patients in remission. These patients were receiving IBD treatment at the time of sample collection. We previously showed that immunosuppressive drugs used to treat IBD such as azathioprine and mercaptopurine inhibited the growth of *C. concisus* strains under laboratory conditions^32^. It is possible that IBD treatment drugs have inhibited the growth of *csep1*-*6bpi*-positive *C. concisus* strains in these patients.

In conclusion, we report an active CD-associated *C. concisus* molecular marker (*csep1*-*6bpi*), which is present in the bacterial chromosome and the novel pICON plasmid. The pathogenic role of the protein encoded by the *csep1*-*6bpi* gene requires further investigation.

## Materials and methods

### Oral *C. concisus* strains used in this study

*C. concisus* strains sequenced in this study were isolated in our previous studies, under the ethics approval granted by the Ethics Committees of the University of New South Wales and the South East Sydney Area Health Service, Australia (HREC 09237/SESIAHS 09/078 and HREC08335/SESIAHS (CHN)07/48)^3,10–12^. Patients and healthy controls were recruited from Sydney, Australia. For the saliva sample from each patient or healthy individual, 12 putative *C. concisus* isolates were collected. The putative *C. concisus* isolates were subjected to a *C. concisus*-specific PCR to confirm the identity of *C. concisus* and then subjected to sodium dodecyl sulphate polyacrylamide gel electrophoresis (SDS-PAGE) for whole-cell protein profile analysis to define the strains. Isolates with identical SDS-PAGE pattern were defined as the same strain. Some individuals were colonised by multiple oral *C. concisus* strains and these strains have been named accordingly^11^. The details of each *C. concisus* strains are listed in Table 1.

### *C. concisus* culture and bacterial DNA extraction

*C. concisus* strains were grown on horse blood agar (HBA) plates under anaerobic conditions supplemented with 5% H_2_ as described previously^35^. Bacterial DNA used for genome sequencing through the MiSeq method was extracted using Gentra Puregene Yeast/Bacteria Kit (Qiagen, Australia) according to manufacturer’s instructions. Bacterial DNA used for genome sequencing through the PacBio method was extracted with phenol-chloroform, followed by purification with Agencourt AMPure XP beads (A63881, Beckman Coulter, UK)^36^. The quality of DNA was determined using Nanodrop and Qubit Fluorometer.

### Genome sequencing, assembly and annotation

The genomes of 37 *C. concisus* strains were sequenced using the MiSeq method at the University of Western Australia, WA, Australia. Bacterial genomic libraries were prepared according to Nextera XT protocol (Ver. May 2012). Libraries were prepared using Nextera XT V2 on MiSeq Personal Sequencer (Illumina Inc., San Diego, CA, USA) running version MiSeq Control Software 1.1.1 to obtain 250 bp paired-end reads. Reagent contamination was controlled by barcoding all DNA samples and primers. The quality of reads was assessed by the Phred quality score and the reads mapping fold coverage was determined with qualimap_v2.2.1^37^. The raw reads were assembled as described previously^23^. Contigs <1000 bp and with coverage <10× were removed. Gene annotation was performed by the Rapid Annotations software at Subsystems Technology server (RAST, Ver. 2.0)^38^.

The draft genome of *C. concisus* strain P2CDO4 has been sequenced in our previous study using the MiSeq method. To confirm the identity of a novel genomic fragment, the DNA extracted from this strain was re-sequenced in this study using the PacBio method to obtain the complete genome. Large insert libraries (20 kb) were constructed and sequenced using the PacBio RS II platform (Ramaciotti Centre for Genomics, University of New South Wales, Australia). The PacBio reads were assembled into contigs using CANU v 1.3^39^. The assembly was rearranged using Circlator to produce accurate linear representations of circular sequences^40^. The assembly was then subjected to polishing using Quiver, followed by polishing with Illumina reads obtained from our previous study using Pilon^23,41,42^.

We ensured that all genomes sequenced using the MiSeq or PacBio methods had fold coverage of at least 50× or 20× respectively, which were shown to be adequate for genome characterisation^39,43^.

### Determination of *C. concisus* genomospecies

The genomospecies status of the 37 *C. concisus* strains sequenced using the MiSeq method in this study was determined by phylogenetic analysis of the core genome and the 23S rRNA gene. The 23S rRNA gene phylogenetic tree was generated using the maximum likelihood method implemented in MEGA6^44^. The core genome phylogenetic tree was generated by Roary^45^.

### Plasmid identification

By comparing the draft genomes of 63 *C. concisus* strains generated using the MiSeq method, we found a genomic fragment that was only present in the draft genomes of strains P2CDO4, P20CDO-S2 and P20CDO-S3, which were oral strains isolated from the two relapsed CD patients with previous ileocecal resection due to small bowel stricture (Table 1). The fragment from these three strains were aligned using Mauve^46^. We re-sequenced the genome of strain P2CDO4 using PacBio method, which generated two contigs with the large contig being the chromosome, and the small contig that corresponds to the genome fragment being the plasmid. The plasmid was also consistently identified by plasmidSPAdes^47^. Plasmid identification was performed using bioinformatics tools according to previously described criteria^27^. The criteria defining a plasmid include the presence of *ori* site containing AT-rich repetitive sequences, *dnaA* box sequences and plasmid-encoded replication initiation protein^27^. The *ori* and the *dnaA* box sequences were predicted using Ori-Finder, and AT-rich repetitive sequences were identified using Tandem Repeats Finder^48,49^. DNAPlotter was used to visualise the plasmid genome^50^.

To examine whether the plasmid identified in *C. concisus* strains P2CDO4, P20CDO-S2 and P20CDO-S3 shared similarities with known plasmids, the nucleotide sequence of the identified plasmid was compared with the bacterial genomes (Taxonomy ID for bacteria: 2) available in NCBI genome database using BLASTn.

These approaches led to the identification of a novel *C. concisus* plasmid, the pICON plasmid (see results section).

### Detection of the pICON plasmid in *C. concisus* strains isolated from patients with IBD and controls

The presence of the pICON plasmid in the 63 oral *C. concisus* strains isolated from patients with IBD and healthy controls was firstly examined by genome search using BLASTn^51^, then confirmed using two PCR methods targeting genes CCS77_2029 and CCS77_2093, which are genes exclusively present in the pICON plasmids not the chromosomes. The primers and thermocycling conditions were listed in Supplementary Figure S6A. The prevalence rates of pICON plasmid in *C. concisus* strains isolated from patients with IBD and controls were compared.

### Prediction of secreted proteins and identification of putative virulence factors in *C. concisus* pICON plasmid

Secreted proteins were predicted using SignalP version 4.0, which identifies signal peptides in queried proteins^52^.

Virulence Factors Database (VFDB) was used for identification of putative virulence factors in the pICON plasmid^53^. The plasmid proteins were queried against the virulence factors in the VFDB core dataset using BLASTp with a cut-off *E*-value of 0.05^54^.

### Detection and comparison of *csep1* genes and Csep1 proteins in *C. concisus* strains isolated from patients with IBD and controls

The presence of the *csep1* gene in the 63 oral *C. concisus* strains isolated from patients with IBD and controls was first examined by genome search using BLASTn. The sequences of the *csep1* gene and Csep1 protein in different *C. concisus* strains were compared using Muscle^55^.

The presence of *csep1* gene in the 63 oral *C. concisus* strains was then confirmed using PCR methods. PCR primers targeting the conserved regions upstream and downstream of *csep1*^P^ (Pfla_F and Pfla_R), *csep1*^C^ (Cfla_F and Cfla_R) and *csep1*^C2^ (C2fla_F and C2fla_R) were designed using Primer-BLAST (Supplementary Figure S6B)^56^. Strains that were negative for *csep1* genes in the above PCR reactions were subjected to an additional PCR detection targeting the conserved regions within the *csep1* genes (csep1_F and csep1_R), which amplifies all three copies of the *csep1* genes (Supplementary Figure S6C). All positive PCR products were sequenced from both ends using BigDye v 3.1 reagents (Applied Biosystems, Foster City, CA) and analysed on an ABI Capillary DNA Sequencer ABI3730 (Applied Biosystems) at Ramaciotti Centre for Genomics.

As mentioned above, the *csep1* gene was found at three different positions within the genome: one in the pICON plasmid and two in the chromosome. To investigate whether specific genomic structures are associated with the insertion site of the *csep1* gene, the flanking genes of *csep1*-negative and positive *C. concisus* strains were compared using BLASTn and visualised using EasyFig^51,57^.

### Phylogenetic analysis of the *csep1* genes in different *C. concisus* strains

The phylogenetic tree of the 26 *csep1* genes from the 63 oral *C. concisus* strains was generated using the maximum likelihood method implemented in MEGA6^44^.

### Detection of the expressed Csep1 proteins

*C. concisus* P2CDO4, which contains the *csep1* gene in both the plasmid pICON and the chromosome, was used to examine the Csep1 protein expression. The strain was cultured on HBA plates for 48 h. Following cultivation, bacteria were collected from the plates and resuspended in 20 ml of heart infusion broth (HIB) (Oxoid^TM^, Australia) to a final OD_600_ of 0.1, and further incubated for 24 h with rotation at 200 rpm^35^.

Following incubation in HIB, both *C. concisus* bacteria and supernatant were collected by centrifugation. The whole-cell lysates were prepared by three freeze-thaw cycles of the bacterial cells. The protein concentrations were determined using a BCA assay kit (Thermo Fisher Scientific, USA), and 20 µg of proteins were loaded onto SDS-polyacrylamide gel and separated by electrophoresis. The culture supernatant from bacteria cultured using HIB was filtered through a 0.22 μm MILLEX GP filter (Merck Millipore Ltd, Ireland) to remove any remaining bacteria. Supernatant was concentrated using Amicon^®^ Ultra 3 K columns (Merck Millipore Ltd, Ireland), which was then loaded onto SDS-polyacrylamide gel and separated by electrophoresis. Protein bands were excised from Coomassie Blue stained polyacrylamide gels and digested with trypsin. Digested peptides were separated by liquid chromatography and analysed using a LTQ-FT Ultra mass spectrometer (Thermo Electron, Bremen, Germany) as previously described^12^. All MS/MS spectra were searched against the NCBI database using MASCOT (version 2.5.1) and then Scaffold Q+ (v.4.7.3, Proteome software, OR, US) was used to validate peptide and protein identities against the proteins encoded on the pICON plasmid of *C. concisus* strain P2CDO4^58^. Mass spectrometry was conducted at the Bioanalytical Mass Spectrometry Facility, University of New South Wales, Australia.

### Genbank sequence submission

The annotated complete genome of *C. concisus* strain P2CDO4 including its pICON plasmid and chromosome was submitted to Genbank genome assembly database (Biosample ID: SAMN07160232; Bioproject ID: PRJNA388128; accession number: CP021642 and CP021643 for chromosome and pICON plasmid respectively). The assembled genomes of the remaining 37 *C. concisus* strains sequenced using the MiSeq method were submitted to Genbank under the Bioproject ID PRJNA388128.

### The presence of *csep1* gene in the genome of other *C. concisus* strains

Currently, there are a further 125 *C. concisus* strains’ genomes available in public databases. We examined the presence of pICON and *csep1* gene in these strains by genome search and comparison of the flanking genes.

### Statistical analysis

Fisher’s exact test (two-tailed) was used to compare the prevalence of pICON plasmid, *csep1* gene in *C. concisus* strains isolated from patients with IBD and healthy controls. Statistical analyses were performed using GraphPad Prism 6 software (San Diego, CA). *P* values <0.05 were considered as statistically significant.

## Electronic supplementary material

Supplementary Table S1

Supplementary Table S2

Supplementary Figure S1

Supplementary Figure S2

Supplementary Figure S3

Supplementary Figure S4

Supplementary Figure S5

Supplementary Figure S6

Supplementary Figure legends

## References

[1] Zhang L (2009). Detection and isolation of *Campylobacter* species other than *C. jejuni* from children with Crohn’s disease. J. Clin. Microbiol..

[2] Mukhopadhya I (2011). Detection of *Campylobacter concisu*s and other* Campylobacter* species in colonic biopsies from adults with ulcerative colitis. PLoS ONE.

[3] Mahendran V (2011). Prevalence of *Campylobacter* species in adult Crohn’s disease and the preferential colonization sites of *Campylobacter* species in the human intestine. PLoS ONE.

[4] Kirk KF (2016). Optimized cultivation of *Campylobacter concisus* from gut mucosal biopsies in inflammatory bowel disease. Gut Pathog..

[5] Sartor RB, Mazmanian SK (2012). Intestinal microbes in inflammatory bowel diseases. Am. J. Gastroenterol..

[6] Lindblom G (1995). *Campylobacter upsaliensis*, *C. sputorum sputorum* and *C. concisus* as common causes of diarrhoea in Swedish children. Scand. J. Infect. Dis..

[7] Kalischuk L, Inglis G (2011). Comparative genotypic and pathogenic examination of *Campylobacter concisus* isolates from diarrheic and non-diarrheic humans. Bmc. Microbiol..

[8] Nielsen, H. et al. High incidence of *Campylobacter concisus* in gastroenteritis in North Jutland, Denmark: a population-based study. *Clin. Microbiol. Infect.* **19**, 445–450 (2013). 10.1111/j.1469-0691.2012.03852.x 22512739

[9] Lastovica AJ, Roux E (2000). Efficient isolation of *Campylobacteria* from stools. J. Clin. Microbiol..

[10] Zhang L (2010). Isolation and detection of *Campylobacter concisus* from saliva of healthy individuals and patients with inflammatory bowel disease. J. Clin. Microbiol..

[11] Mahendran V (2013). The prevalence and polymorphisms of zonula occluden toxin gene in multiple *Campylobacter concisus* strains isolated from saliva of patients with inflammatory bowel disease and controls. PLoS ONE.

[12] Ismail Y (2012). Investigation of the enteric pathogenic potential of oral *Campylobacter concisus* strains isolated from patients with inflammatory bowel disease. PLoS ONE.

[13] Zhang L (2014). *Campylobacter concisus* and inflammatory bowel disease. World J. Gastroenterol..

[14] Zhang L (2015). Oral *Campylobacter* species: Initiators of a subgroup of inflammatory bowel disease?. World J. Gastroenterol..

[15] Ismail Y (2013). The effects of oral and enteric *Campylobacter concisus* strains on expression of TLR4, MD-2, TLR2, TLR5 and COX-2 in HT-29 cells. PLoS ONE.

[16] Aabenhus R (2005). Delineation of *Campylobacter concisus* genomospecies by amplified fragment length polymorphism analysis and correlation of results with clinical data. J. Clin. Microbiol..

[17] Miller WG (2012). Multilocus sequence typing methods for the emerging *Campylobacter* Species *C. hyointestinalis*, *C. lanienae*, *C. sputorum*, *C. concisus*, and *C. curvus*. Front. Cell Infect. Microbiol..

[18] Mahendran V (2015). Delineation of genetic relatedness and population structure of oral and enteric *Campylobacter concisus* strains by analysis of housekeeping genes. Microbiology.

[19] Engberg J (2005). *Campylobacter concisus*: an evaluation of certain phenotypic and genotypic characteristics. Clin. Microbiol. Infect..

[20] Istivan T. *Molecular Characterisation of Campylobacter concisus: A Potential Etiological Agent of Gastroenteritis in Children* (School of Applied Sciences, RMIT University, Melbourne, 2005).

[21] On S (2013). Characterisation of *Campylobacter concisus* strains from South Africa using amplified fragment length polymorphism (AFLP) profiling and a genomospecies-specific polymerase chain reaction (PCR) assay: identification of novel genomospecies and correlation with clinical data. Afr. J. Microbiol. Res..

[22] Nielsen HL, Nielsen H, Torpdahl M (2016). Multilocus sequence typing of *Campylobacter concisus* from Danish diarrheic patients. Gut Pathog..

[23] Chung HK (2016). Genome analysis of *Campylobacter concisus* strains from patients with inflammatory bowel disease and gastroenteritis provides new insights into pathogenicity. Sci. Rep..

[24] Istivan TS (2004). Characterization of a haemolytic phospholipase A(2) activity in clinical isolates of *Campylobacter concisus*. J. Med. Microbiol..

[25] Mahendran V (2016). Examination of the effects of *Campylobacter concisus* zonula occludens toxin on intestinal epithelial cells and macrophages. Gut Pathog..

[26] Liu F (2016). Zonula occludens toxins and their prophages in *Campylobacter* species. Gut Pathog..

[27] Del Solar G (1998). Replication and control of circular bacterial plasmids. Microbiol. Mol. Biol. Rev..

[28] Deshpande NP (2013). Comparative genomics of *Campylobacter concisus* isolates reveals genetic diversity and provides insights into disease association. BMC Genom..

[29] Huq M (2017). The ribosomal RNA operon (rrn) of *Campylobacter concisus* supports molecular typing to genomospecies level. Gene Rep..

[30] Cornelius AJ (2017). Complete genome sequence of *Campylobacter concisus* ATCC 33237T and draft genome sequences for an additional eight well-characterized *C. concisus* strains. Genome Announc..

[31] Kirk KF (2018). Molecular epidemiology and comparative genomics of *Campylobacter concisus* strains from saliva, faeces and gut mucosal biopsies in inflammatory bowel disease. Sci. Rep..

[32] Liu F (2017). Azathioprine, mercaptopurine, and 5-aminosalicylic acid affect the growth of IBD-associated *Campylobacter* species and other enteric microbes. Front. Microbiol..

[33] Fries, B. C. & Varshney, A. K. Bacterial toxins—Staphylococcal enterotoxin B. *Microbiol. Spectr.* **1**, AID-0002-2012 (2013). 10.1128/microbiolspec.AID-0002-2012 PMC5086421 26184960

[34] Wang Y (2017). *Campylobacter concisus* genomospecies 2 is better adapted to the human gastrointestinal tract as compared with *Campylobacter concisus* genomospecies 1. Front. Physiol..

[35] Lee H (2014). Examination of the anaerobic growth of *Campylobacter concisus* strains. Int. J. Microbiol..

[36] Wilson, K. Preparation of genomic DNA from bacteria. *Curr. Protoc. Mol. Biol.* **Chapter 2**, Unit 2.4 (2001). 10.1002/0471142727.mb0204s56 18265184

[37] Okonechnikov K, Conesa A, García-Alcalde F (2015). Qualimap 2: advanced multi-sample quality control for high-throughput sequencing data. Bioinformatics.

[38] Aziz RK (2008). The RAST Server: rapid annotations using subsystems technology. BMC Genom..

[39] Koren S (2017). Canu: scalable and accurate long-read assembly via adaptive k-mer weighting and repeat separation. Genome Res..

[40] Hunt M (2015). Circlator: automated circularization of genome assemblies using long sequencing reads. Genome Biol..

[41] Walker BJ (2014). Pilon: an integrated tool for comprehensive microbial variant detection and genome assembly improvement. PLoS ONE.

[42] Chin CS (2013). Nonhybrid, finished microbial genome assemblies from long-read SMRT sequencing data. Nat. Methods.

[43] Desai A (2013). Identification of optimum sequencing depth especially for de novo genome assembly of small genomes using next generation sequencing data. PLoS ONE.

[44] Tamura K (2013). MEGA6: molecular evolutionary genetics analysis version 6.0. Mol. Biol. Evol..

[45] Page AJ (2015). Roary: rapid large-scale prokaryote pan genome analysis. Bioinformatics.

[46] Darling AC (2004). Mauve: multiple alignment of conserved genomic sequence with rearrangements. Genome Res..

[47] Antipov, D. et al. plasmidSPAdes: assembling plasmids from whole genome sequencing data. *Bioinformatics* **32**, 3380–3387 (2016). 10.1093/bioinformatics/btw493 27466620

[48] Benson G (1999). Tandem repeats finder: a program to analyze DNA sequences. Nucleic Acids Res..

[49] Gao F, Zhang CT (2008). Ori-Finder: a web-based system for finding *oriC* s in unannotated bacterial genomes. BMC Bioinformatics.

[50] Carver T (2009). DNAPlotter: circular and linear interactive genome visualization. Bioinformatics.

[51] Camacho C (2009). BLAST+: architecture and applications. BMC Bioinformatics.

[52] Petersen TN (2011). SignalP 4.0: discriminating signal peptides from transmembrane regions. Nat. Methods.

[53] Chen L (2016). VFDB 2016: hierarchical and refined dataset for big data analysis–10 years on. Nucleic Acids Res..

[54] Weston J (2005). Semi-supervised protein classification using cluster kernels. Bioinformatics.

[55] Edgar RC (2004). MUSCLE: multiple sequence alignment with high accuracy and high throughput. Nucleic Acids Res..

[56] Ye J (2012). Primer-BLAST: a tool to design target-specific primers for polymerase chain reaction. BMC Bioinformatics.

[57] Sullivan MJ, Petty NK, Beatson SA (2011). Easyfig: a genome comparison visualizer. Bioinformatics.

[58] Searle BC (2010). Scaffold: a bioinformatic tool for validating MS/MS‐based proteomic studies. Proteomics.

