# Supplementary material for: Genomic analysis of oral Campylobacter concisus strains identified a potential bacterial molecular marker associated with active Crohn’s disease
Source: Emerg Microbes Infect. 2018 Apr 11;7:64. doi: 10.1038/s41426-018-0065-6 (PMC5893538; doi:10.1038/s41426-018-0065-6)
Supplement: Supplementary file 1 — Supplementary Table S1 [file 41426_2018_65_MOESM1_ESM.docx]

**Supplementary Table S1. Potential virulence proteins in *C. concisus* pICON plasmids**

| pICON protein | Locus tag | Plasmid protein size (AA) | Virulence protein | Virulence protein size (AA) | Bacteria | E-value | Homology |
| --- | --- | --- | --- | --- | --- | --- | --- |
| Hypothetical protein | CCS77_2035 | 367 | Signal transduction histidine kinase | 339 | *Campylobacter jejuni subsp. jejuni* | 5E-07 | 49% (272AA) (62-333) |
| Hypothetical protein | CCS77_2037 | 171 | Toxin B | 2366 | *Clostridium difficile* | 5E-06 | 46% (206AA) (24-229) |
| DNA-directed RNA polymerase | CCS77_2040 | 2458 | Flagellar hook-length control protein FliK | 598 | *C. jejuni subsp. jejuni* | 2E-05 | 40% (407AA) (60-466) |
| Hypothetical protein | CCS77_2044 | 85 | Motility accessory factor PseD | 653 | *C. jejuni subsp. jejuni* | 0.009 | 44% (75AA) (480-554) |
| Integrase | CCS77_2045 | 329 | Type 1 fimbriae regulatory protein fimE | 198 | *Escherichia coli* | 2E-06 | 48% (145AA) (27-171) |
| Hypothetical protein | CCS77_2050 | 477 | Dot/Icm T4SS effector VpdC | 884 | *L. pneumophila subsp. pneumophila* | 0.04 | 40% (253AA) (448-700) |
| DNA helicase | CCS77_2051 | 609 | Dot/Icm T4SS effector Lem20 | 590 | *L. pneumophila subsp. pneumophila* | 0.003 | 42% (284AA) (38-321) |
| Serine/threonine-protein kinase HipA | CCS77_2052 | 418 | Dot/Icm T4SS effector hypothetical | 312 | *L. pneumophila subsp. pneumophila* | 6E-06 | 45% (202AA) (82-283) |
| Hypothetical protein | CCS77_2055 | 178 | Choline binding protein E | 627 | *Streptococcus pneumoniae* | 0.028 | 50% (92AA) (118-209) |
| IcmO (DotL) protein | CCS77_2057 | 799 | Dot/Icm T4SS coupling protein IcmO/DotL | 783 | *L. pneumophila subsp. pneumophila* | 4E-17 | 53% (163AA) (335-497) |
| Hypothetical protein | CCS77_2062 | 962 | Type VII secretion system protein EsaA | 1009 | *Staphylococcus aureus subsp. aureus* | 0.016 | 43% (212AA) (139-350) |
| Single-stranded DNA specific exonuclease RecJ | CCS77_2063 | 663 | Flagellar hook-length control protein FliK | 598 | *C. jejuni subsp. jejuni* | 0.031 | 45% (133AA) (316-448) |
| Hypothetical protein | CCS77_2065 | 909 | Exo-alpha-sialidase | 594 | *Clostridium perfringens* | 0.005 | 47% (100AA) (85-184) |
| Hypothetical protein | CCS77_2071 | 123 | EHEC factor for adherence | 3223 | *Escherichia coli* | 0.019 | 47% (106AA) (2575-2680) |
| Hypothetical protein^#^ | CCS77_2072 | 524 | Choline binding protein A | 693 | *S. pneumoniae* | 0.008 | 37% (210AA) (63-272) |
| **Csep1^P #^** | **CCS77_2074** | **222** | **Staphylococcal enterotoxin B** | **266** | ***S. aureus*** | **0.04** | **42% (105AA) (55-159)** |
| Hypothetical protein^#^ | CCS77_2082 | 475 | T4SS protein VirB4 | 822 | *C. jejuni subsp. jejuni* | 0.028 | 53% (77AA) (526-602) |
| Hypothetical protein | CCS77_2091 | 201 | Sugar transferase | 776 | *C. jejuni subsp. jejuni* | 0.034 | 44% (119AA) (342-460) |
| Hypothetical protein | CCS77_2093 | 117 | Staphylococcal enterotoxin H precursor | 241 | *S. aureus subsp. aureus* | 0.008 | 55% (76AA) (164-239) |
| Hypothetical protein | CCS77_2095 | 528 | Putative 3-ketoacyl-ACP synthase CylI | 731 | *Streptococcus agalactiae* | 0.006 | 54% (50AA) (21-70) |
| Hypothetical protein | CCS77_2103 | 715 | Protein P216 | 1879 | *Mycoplasma hyopneumoniae* | 0.009 | 65% (109AA) (1129-1237) |
| Hypothetical protein | CCS77_2104 | 648 | Dot/Icm T4SS effector LegC2/YlfB | 405 | *L. pneumophila subsp. pneumophila* | 0.001 | 45% (176AA) (178-353) |
| Hypothetical protein | CCS77_2113 | 225 | Type VII secretion system protein EssA | 152 | *S. aureus subsp. aureus* | 0.013 | 51% (73AA) (22-94) |
| Hypothetical protein | CCS77_2115 | 534 | Dot/Icm T4SS effector LegK2 | 538 | *L. pneumophila subsp. pneumophila* | 0.032 | 42% (252AA) (181-432) |
| Plasmid replication initiation protein | CCS77_2118 | 340 | Cytotoxin | 3169 | *Escherichia coli* | 0.041 | 48% (89AA) (518-606) |
| Hypothetical protein | CCS77_2129 | 135 | Staphylokinase precursor | 163 | *S. aureus subsp. aureus* | 0.013 | 50% (54AA) (22-75) |

Proteins encoded by pICON plasmids in *C. concisus* P2CDO4, P20CDO-S2 and P20CDO-S3 were compared with known virulence factors in the virulence factor database. Virulence proteins included are those having E-values less than 0.05 when compared with *C. concisus* proteins, and for virulence proteins that shared homology with the same *C. concisus* protein from multiple strains, the lowest E-values were noted. The protein encoded by CCS77_2074 was found to share homology with Staphylococcal enterotoxin B (bolded). The homology of putative virulence proteins in pICON plasmids to known bacterial virulence proteins based on BLASTp was expressed as % similarity (the number of amino acids used for comparison) (the start and end positions of the known bacterial virulence proteins that matched). Csep1^P^ was detected in the bacterial culture supernatant of *C. concisus* P2CDO4 using mass spectrometry. ^#^Proteins predicted to contain signal peptide. AA: amino acid. T4SS: type IV secretion system. Csep1^P^: pICON plasmid encoded Csep1 protein.
