# Supplementary material for: Genomic analysis of oral Campylobacter concisus strains identified a potential bacterial molecular marker associated with active Crohn’s disease
Source: Emerg Microbes Infect. 2018 Apr 11;7:64. doi: 10.1038/s41426-018-0065-6 (PMC5893538; doi:10.1038/s41426-018-0065-6)
Supplement: Supplementary file 2 — Supplementary Table S2 [file 41426_2018_65_MOESM2_ESM.docx]

**Supplementary Table S2.** **pICON plasmid proteins predicted to be secreted**

| **Locus tag** | **Protein name** |
| --- | --- |
| CCS77_2072 | hypothetical protein |
| CCS77_2073 | hypothetical protein |
| CCS77_2074 | Csep1^P^ |
| CCS77_2075 | hypothetical protein |
| CCS77_2078 | hypothetical protein |
| CCS77_2082 | hypothetical protein |
| CCS77_2083 | hypothetical protein |
| CCS77_2084 | hypothetical protein |
| CCS77_2087 | hypothetical protein |
| CCS77_2096 | hypothetical protein |
| CCS77_2099 | hypothetical protein |
| CCS77_2102 | Micrococcal nuclease-like protein |

Prediction of the presence of signal peptide was performed using SignalP.
