# Supplementary figures and images for: Genomic analysis of oral Campylobacter concisus strains identified a potential bacterial molecular marker associated with active Crohn’s disease
Source: Emerg Microbes Infect. 2018 Apr 11;7:64. doi: 10.1038/s41426-018-0065-6 (PMC5893538; doi:10.1038/s41426-018-0065-6)

Supplementary Figure S1

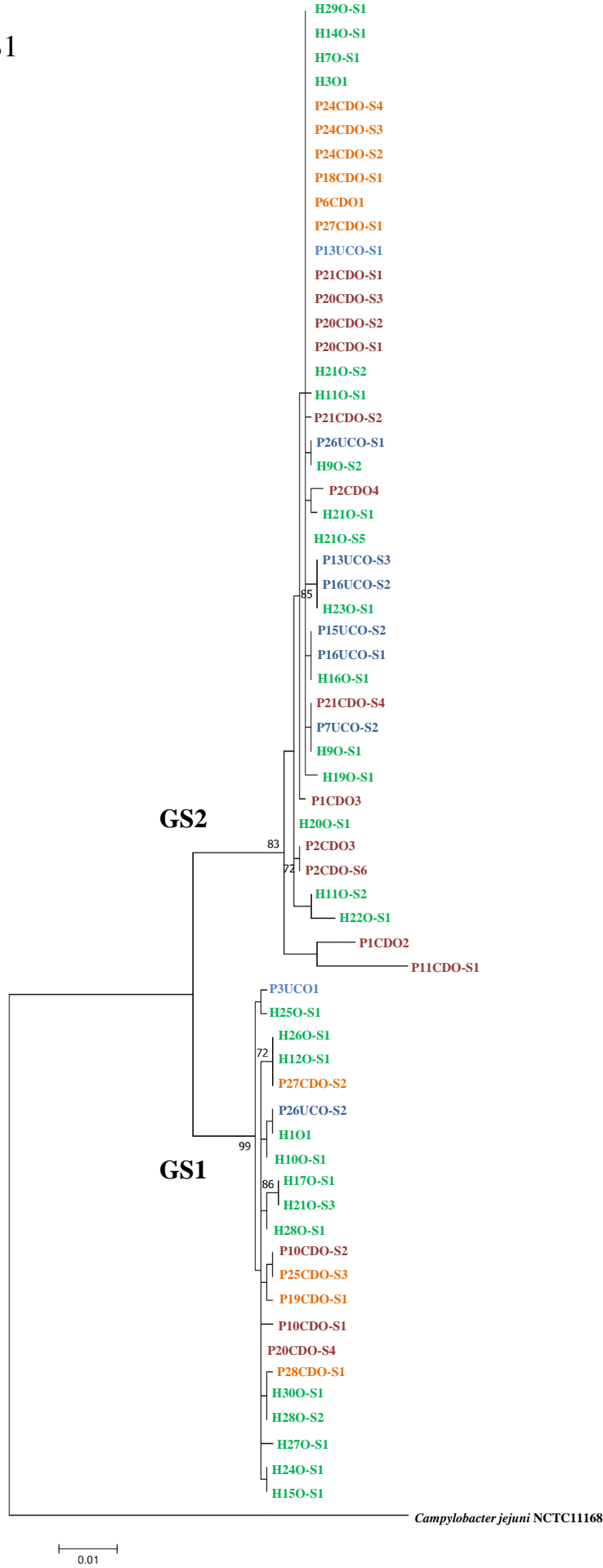

Supplement: Supplementary file 3 — Supplementary Figure S1 [file 41426_2018_65_MOESM3_ESM.pdf]

Supplementary Figure S2

A

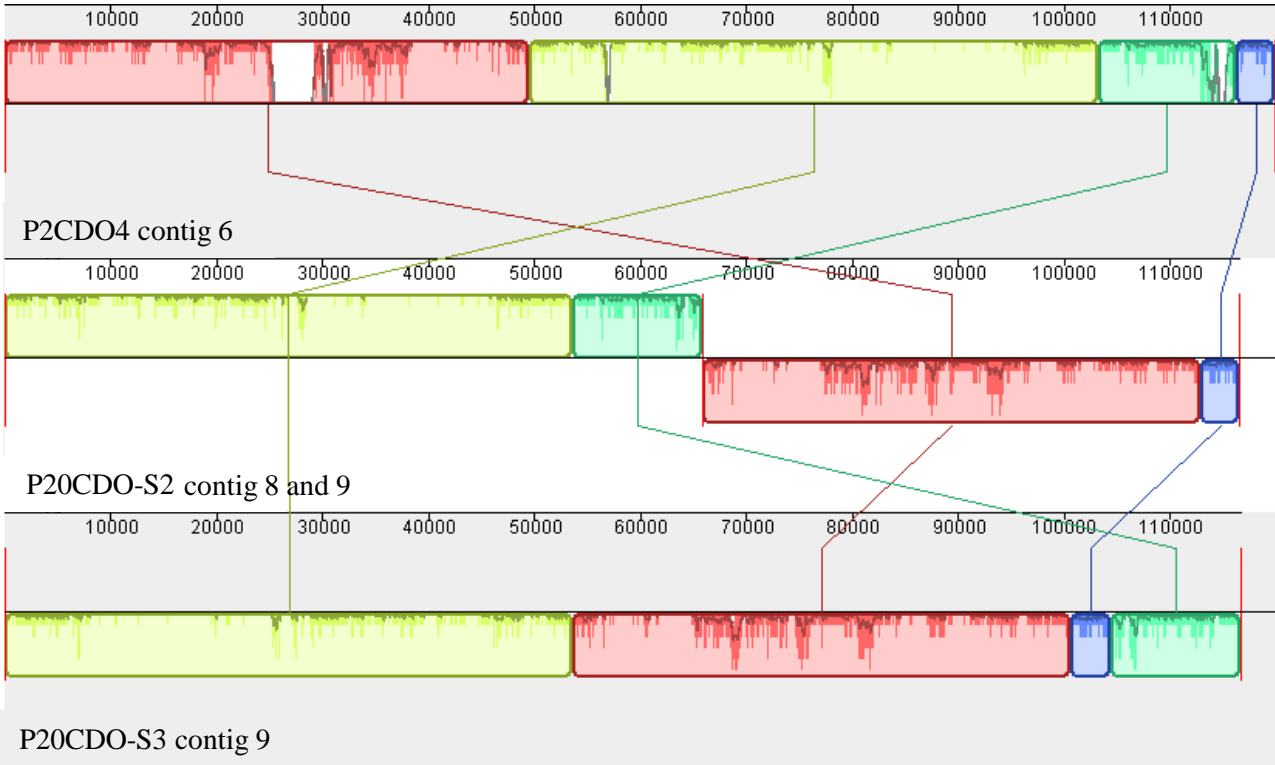

B

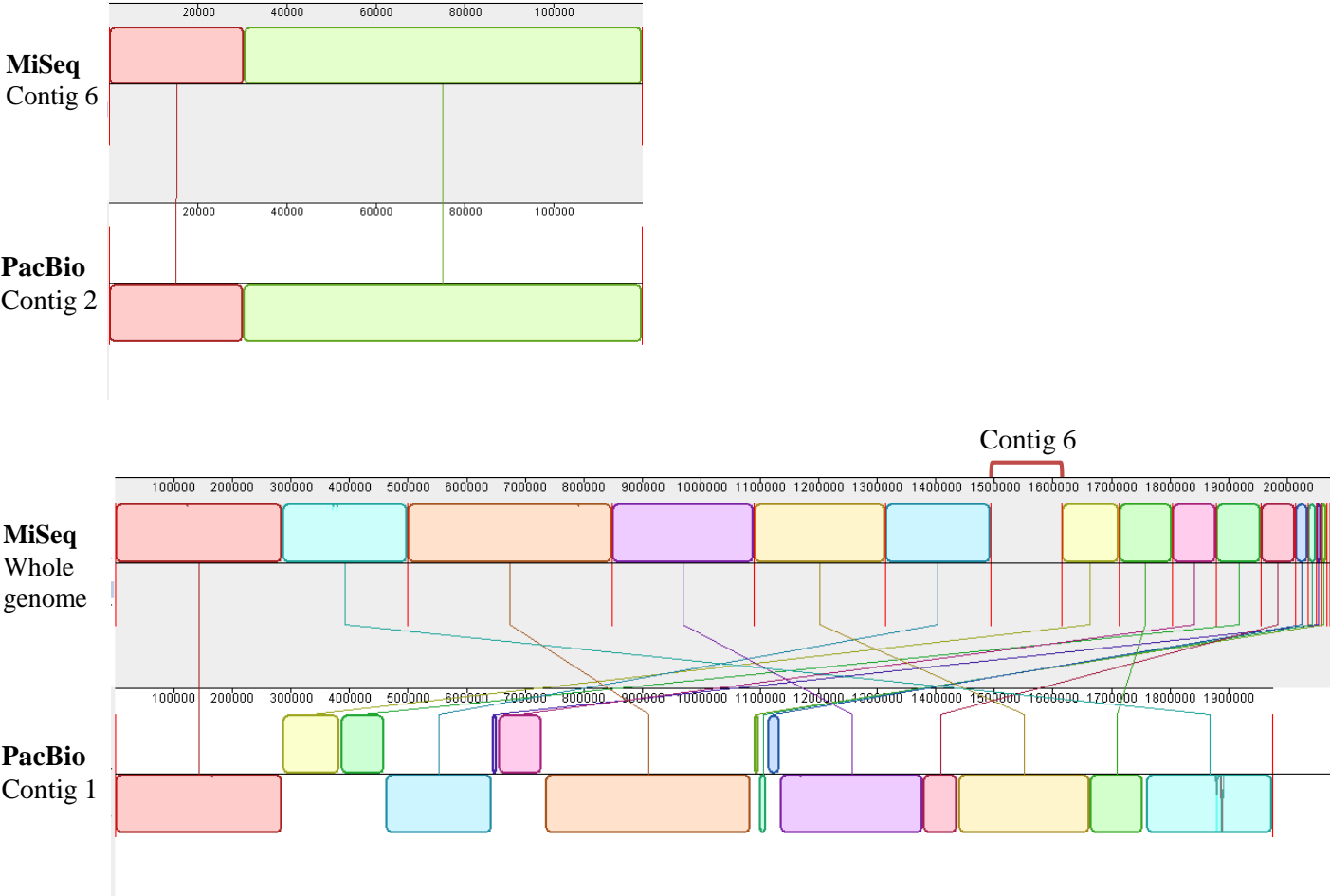

Supplement: Supplementary file 4 — Supplementary Figure S2 [file 41426_2018_65_MOESM4_ESM.pdf]

Supplementary Figure S5

A

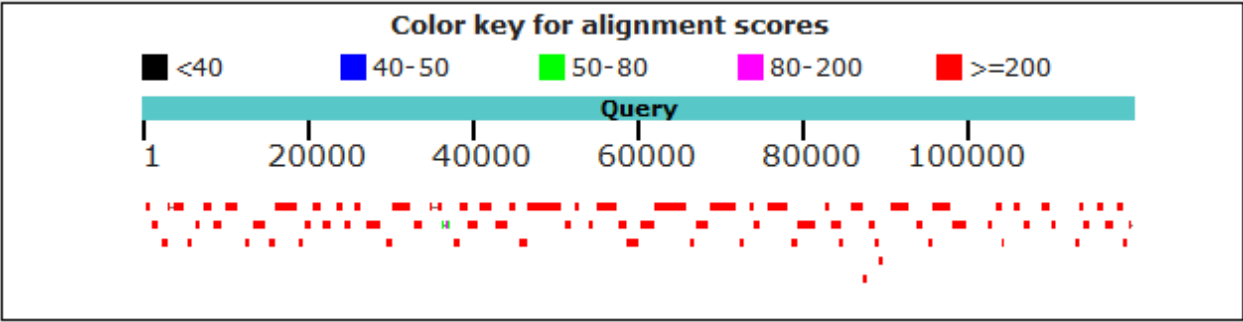

B

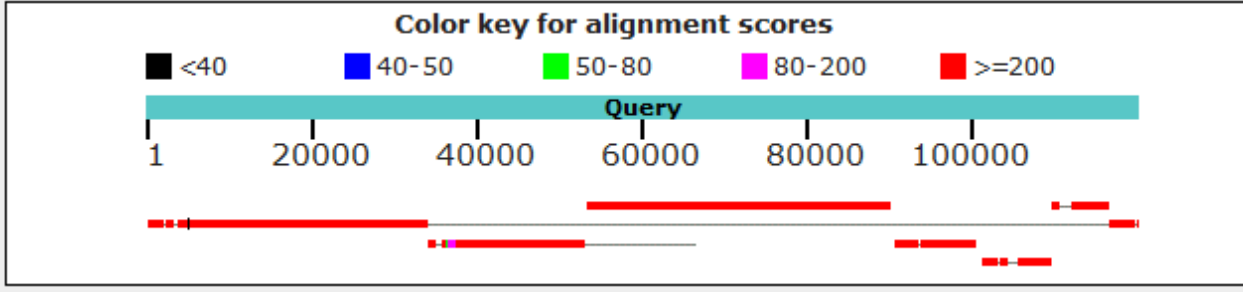

Supplement: Supplementary file 7 — Supplementary Figure S5 [file 41426_2018_65_MOESM7_ESM.pdf]
