# Supplementary material for: Genomic analysis of oral Campylobacter concisus strains identified a potential bacterial molecular marker associated with active Crohn’s disease
Source: Emerg Microbes Infect. 2018 Apr 11;7:64. doi: 10.1038/s41426-018-0065-6 (PMC5893538; doi:10.1038/s41426-018-0065-6)
Supplement: Supplementary file 5 — Supplementary Figure S3 [file 41426_2018_65_MOESM5_ESM.pdf]

Supplementary Figure S3

```
P2CDO4_csep1P              ATGAAAAAGCAAATTTTAGCTTCAGTCTTAGCCAGCGTGTTGGCCACAAGTAGCGTATAT 60
P13UCO-S3_csep1P           ATGAAAAAGCAAATTTTAGCTTCAGTCTTAGCCAGCGTGTTGGCCACAAGTAGCGTATAT 60
P26UCO-S1_csep1C           ATGAAAAAGCAAATTTTAGCTTCAGTATTAGCCAGCGTGTTAGCCACAAGTAGCGTATAT 60
P2CDO4_csep1C              ATGAAAAAGCAAATTTTAGCTTCAGTCTTAGCCAGTGTGTTAGCCATAAGTAGCGTATAT 60
P20CDO-S2_csep1P           ATGAAAAAGCAAATTTTAGCTTCAGTCTTAGCCAGCGTGTTGGCCACAAGTAGCGTATAT 60
P20CDO-S3_csep1P           ATGAAAAAGCAAATTTTAGCTTCAGTCTTAGCCAGCGTGTTGGCCACAAGTAGCGTATAT 60
P16UCO-S2_csep1C           ATGAAAAAGCAAATTTTAGCTTCAGTCTTAGTTGGCGCTTTGGCTGTAAGTAGTGCTTCA 60
H11O-S2_csep1C             ATGAAAAAGCAAATTTTAGCTTCAGTCTTAGCTAGCGTGTTGGCCACAAGTAGCGTATAT 60
P11CDO-S1_csep1C           ATGAAAAAGCAAATTTTAGCTTCAGTATTAGCCAGCGTGTTGGCCACAAGTAGCGTATAT 60
P7UCO-S2_csep1C            ATGAAAAAGCAAATTTTAGCTTCAGTCTTAGCCAGCGCGTTGGCCACAAGTAGCGTATAT 60
H9O-S1_csep1C              ATGAAAAAGCAAATTTTAGCTTCAGTCTTAGCCAGCGCGTTGGCCACAAGTAGCGTATAT 60
H20O-S1_csep1C             ATGAAAAAGCAAATTTTAGCTTCAGTCTTAGCTAGCGTGTTGGCCACAAGTAGCGTATAT 60
P18CDO-S1_csep1C           ATGAAAAAGCAAATTTTAGCTTCAGTCTTAGCCAGCGTTTTAGCCACAAGTAGCGTATAT 60
H9O-S2_csep1C              ATGAAAAAGCAAATTTTAGCTTCTATTCTAGTTGGTGCGCTTGCTGTAAGTAGTGCTTCA 60
P13UCO-S1_csep1C           ATGAAAAAGCAAATTTTAGCTTCAGTCTTAGCCAGCGTGTTAGCCACAAGTAGCGTATAT 60
P27CDO-S1_csep1C           ATGAAAAAGCAAATTTTAGCTTCAGTCTTAGCCAGCGTGTTAGCCACAAGTAGCGTATAT 60
H29O-S1_csep1C             ATGAAAAAGCAAATTTTAGCTTCAGTCTTAGCCAGCGTGTTAGCCACAAGTAGCGTATAT 60
P2CDO3_csep1C              ATGAAAAAGCAAATTTTAGCTTCAGTCTTAGCCAGCGTGTTGGCCACAAGTAGCGTATAT 60
P2CDO-S6_csep1C            ATGAAAAAGCAAATTTTAGCTTCAGTCTTAGCCAGCGTGTTGGCCACAAGTAGCGTATAT 60
P1CDO3_csep1C              ATGAAAAAGCAAATTTTAGCTTCTGTTCTAGTTGGCGCTTTGACTGCAGGTAGTGCTTCA 60
P20CDO-S1_csep1C           ATGAAAAAGCAAATTTTAGCTTCAATCTTAGCCAGCGTGTTGGCCACAAGTAGCGTATAT 60
P20CDO-S3_csep1C           ATGAAAAAGCAAATTTTAGCTTCAATCTTAGCCAGCGTGTTGGCCACAAGTAGCGTATAT 60
H3O1_csep1C                ATGAAAAAGCAAATTTTAGCTTCAGTCTTAGCCAGCGTGTTGGCCACAAGTAGCGTATAT 60
P11CDO-S1_csep1C2          ATGAAAAAGCAAATTTTAGCTTCAGTATTAGCCAGCGTGTTGGCCACAAGTAGCGTATAT 60
P21CDO-S2_csep1C           ATGAAAAAGCAAATTTTAGCTTCAGTCTTAGCCAGTGTGTTAGCCATAAGTAGCGTATAT 60
H21O-S1_csep1C             ATGAAAAAGCAAATTTTAGCTTCAGTCTTAGCCAGCGTTTTAGCCACAAGTAGCGTATAT 60
                           ***********************  *  ***   * *   *  *   * **** *  *

                                     A     B
P2CDO4_csep1P              GCTTGGGGC---TTTGGAGGAAGCAGTAAAGAAAAGCCAAATTATGATTATAGCGCTGAT 117
P13UCO-S3_csep1P           GCTTGGGGC---TTTGGAGGAAGTAGTAAAGAGAAACCAAATTATGATTATAGCGCTGAT 117
P26UCO-S1_csep1C           GCTTGGGGC---TTT---GGGAGTAGTAGAGAAAAGCCAAATTATGATTATAGCGCTGAT 114
P2CDO4_csep1C              GCTTGGGGC---TTT---GGAAGTAGTAGAGAAAAGCCTAACTATGATTATAGTGGGGAG 114
P20CDO-S2_csep1P           GCTTGGGGT---TTT---GGAAGTAGTAAAGAAAAACCAAATTACGATTATAGTGGGGAG 114
P20CDO-S3_csep1P           GCTTGGGGT---TTT---GGAAGTAGTAAAGAAAAACCAAATTACGATTATAGTGGGGAG 114
P16UCO-S2_csep1C           GCTTGGAGTTTGTTT---GGCGACGATAGAGAAAAGCCAAATTATGATTATAGTGGGGAG 117
H11O-S2_csep1C             GCTTGGGGC---TTT---GGGAGTAGCAAAGAAAAACCAAATTATGATTATAGTGGGGAT 114
P11CDO-S1_csep1C           GCTTGGGGC---TTT---GGGAGTAGCAAAGAAAAACCAAATTATGATTATAGTGGGGAT 114
P7UCO-S2_csep1C            GCTTGGGGC---TTT---GGGAGTAGCAAAGAAAAGCCAAATTATGATTATAGTGGGGAT 114
H9O-S1_csep1C              GCTTGGGGC---TTT---GGGAGTAGCAAAGAAAAGCCAAATTATGATTATAGTGGGGAT 114
H20O-S1_csep1C             GCTTGGGGC---TTTGGAGGAAGTAGTAAAGAAAAGCCTAATTATGATTATAGTGGCGAA 117
P18CDO-S1_csep1C           GCTTGGGGC---TTT---GGAAGTAGTAGAGAAAAGCCAAATTATGATTATAGCGCTGAT 114
H9O-S2_csep1C              GCCTGGAGTTTGTTT---GGCGGAGAAAGAGAAAAGCCAAATTATGATTATAGCGCTGAT 117
P13UCO-S1_csep1C           GCTTGGGGC---TTT---GGGAGTAGTAGAGAAAAGCCAAATTATGATTATAGCGCCGAT 114
P27CDO-S1_csep1C           GCTTGGGGC---TTT---GGGAGTAGTAGAGAAAAGCCAAATTATGATTATAGCGCCGAT 114
H29O-S1_csep1C             GCTTGGGGC---TTT---GGGAGTAGTAGAGAAAAGCCAAATTATGATTATAGCGCCGAT 114
P2CDO3_csep1C              GCTTGGGGC---TTT---GGGAGTAGCAAAGAAAAGCCAAATTATGATTATAGTGGGGAG 114
P2CDO-S6_csep1C            GCTTGGGGC---TTT---GGGAGTAGCAAAGAAAAGCCAAATTATGATTATAGTGGGGAG 114
P1CDO3_csep1C              GCTTGGAGTTTGTTT---GGCGGCGATAGAGAAAAGCCAAATTATGATTATAGTGGGGAG 117
P20CDO-S1_csep1C           GCTTGGGGC---TTT---GGGAGTAGTAGAGAAAAGCCAAATTATGATTATAGTGGGGAG 114
P20CDO-S3_csep1C           GCTTGGGGC---TTT---GGGAGTAGTAGAGAAAAGCCAAATTATGATTATAGTGGGGAG 114
H3O1_csep1C                GCTTGGGGC---TTTGGAGGAAGTAGTAAAGAGAAACCAAATTATGATTATAGTGGGGAG 117
P11CDO-S1_csep1C2          GCTTGGGGC---TTTGGAGGAAGTAGTAAAGAAAAGCCAAATTATGATTATAGTGGAGAG 117
P21CDO-S2_csep1C           GCTTGGGGC---TTT---GGAAGTAGTAGAGAAAAGCCTAACTATGATTATAGTGGGGAG 114
H21O-S1_csep1C             GCTTGGGGC---TTTGGAGGAAGTAGTAGAGAAAAGCCAAATTATGATTATAGTGGGGAG 117
                           ** *** *    ***   **       * *** ** ** ** ** ******** *  **
```

Supplementary Figure S3

```
                                                  C
P2CDO4_csep1P         AAAAAGCTTATAGAAATAAATACT---------AGACCAATTAGCACTGACAATGCAAAA 168
P13UCO-S3_csep1P      AAAAAGCTTATAGAAATAAATACT---------AGGCCAATTAGCACTGACAATGCAAAA 168
P26UCO-S1_csep1C      AAAAAGCTTATAGAAATAAATACT---------AGACCGATTAGCACTGATAATGCGAAA 165
P2CDO4_csep1C         AAAGAATTTATTCAGAAAAATACT---------AAAGAAATTGGCGGTGACAATGCTAAA 165
P20CDO-S2_csep1P      AAAGAATTTATTCAGAAAAATACT---------AAAGAAATTGGCGGTGACAATGCTAAA 165
P20CDO-S3_csep1P      AAAGAATTTATTCAGAAAAATACT---------AAAGAAATTGGCGGTGACAATGCTAAA 165
P16UCO-S2_csep1C      AAAGAATTCATTCAGAAAAATACT---------CAAAAGCTTGGGACTAATGATGCAAAA 168
H11O-S2_csep1C        AAACAGTTTATAGAAACTAACACG---------AAAAAAATAGACACAGATAGGGCAAAA 165
P11CDO-S1_csep1C      AAACAGTTTATAGAAACTAACACG---------AAAAAAATAGACACAGATAGGGCAAAA 165
P7UCO-S2_csep1C       AAACAGTTTATAGAAACTAACACG---------AAAAAAATAGACACAGATAGGGCAAAA 165
H9O-S1_csep1C         AAACAGTTTATAGAAACTAACACG---------AAAAAAATAGACACAGATAGGGCAAAA 165
H20O-S1_csep1C        AAAGAATTTATTCAGAAAAATACT---------CAAAAGCTTGGGACTAATGACGCAAAA 168
P18CDO-S1_csep1C      AAAAAGCTTATAGAAACCAATACA---------CAAAAAATTGGCACTGAAAAGCAAAA 165
H9O-S2_csep1C         AAAAAGCTTATAGAAACCAATACA---------CAAAAAATTGGCACTGAAAAGCAAAA 168
P13UCO-S1_csep1C      AAAAAGCTTATAGAAACCAATACA---------CAAAAAATTGGCACTGAAAAGCAAAA 165
P27CDO-S1_csep1C      AAAAAGCTTATAGAAACCAATACA---------CAAAAAATTGGCACTGAAAAGCAAAA 165
H29O-S1_csep1C        AAAAAGCTTATAGAAACCAATACA---------CAAAAAATTGGCACTGAAAAGCAAAA 165
P2CDO3_csep1C         AAAGAGCTAATGTCATTAAGTTCTTTCTCAGACAAAGATCTGCGAATTGATGATGCAAAA 174
P2CDO-S6_csep1C       AAAGAGCTAATGTCATTAAGTTCTTTCTCAGACAAAGATCTGCGAATTGATGATGCAAAA 174
P1CDO3_csep1C         AAAGAGCTAATGTCATTAAGTTCTTTTTCAGACAAAGATCTGCGAATTGATGATGCAAAA 177
P20CDO-S1_csep1C      AAAGAGCTAATGTCATTAAGTTCTTTCTCAGACAAAGATCTGCGAATTGATGATGCAAAA 174
P20CDO-S3_csep1C      AAAGAGCTAATGTCATTAAGTTCTTTCTCAGACAAAGATCTGCGAATTGATGATGCAAAA 174
H301_csep1C           AAAGAGCTAATGTCATTAAGTTCTTTCTCGGACAAAGATCTGCGAATTGATGATGCAAAA 177
P11CDO-S1_csep1C2     AAAGAGCTAATGTCATTAAGTTCTTTTTCAGGAAATAATCTACGAATTGATGATGCAAAA 177
P21CDO-S2_csep1C      AAAGAGCTAATGTCATTAAGTTCTTTTTCAGACAAAGATCTGCGAATTGATGATGCAAAA 174
H21O-S1_csep1C        AAAGAGCTAATGTCATTAAGTTCTTTCTCAGACAAAGATCTGCGAATTGATGATGCAAAA 177
                      *** *  * **       *   *                 *        *    ** ***

P2CDO4_csep1P         TATTGGTGTTTTGACAAAGGTAATGATTTAGGTTGTCTTTCGCTAGAAAAACTAGAAGCT 228
P13UCO-S3_csep1P      TATTGGTGTTTTGACAAAGGTAATGATTTAGGTTGTCTTTCGCTAGAAAAACTAGAAGCT 228
P26UCO-S1_csep1C      TATTGGTGTTTTGATAAAGGCAATGATTTAGGTTGTCTTTCGCTAGAAAAATTAGAAGCT 225
P2CDO4_csep1C         TACTGGTGCTTTGATAAAGGCAATGATCTAGGTTGTCTTTCGCTAGAAAAATTAGAAGCT 225
P20CDO-S2_csep1P      TACTGGTGCTTTGATAAAGGCAATGATCTAGGTTGTCTTTCGCTAGAAAAATTAGAAGCT 225
P20CDO-S3_csep1P      TACTGGTGCTTTGATAAAGGCAATGATCTAGGTTGTCTTTCGCTAGAAAAATTAGAAGCT 225
P16UCO-S2_csep1C      AGATGGTGCTTTGATAAAGGCAACGATCTAGGTTGTCTTTGGCTAGAAAAATTAGAGGCT 228
H11O-S2_csep1C        TATTGGTGTTTAGATCAAGGTAATGATCTAGGCTGCCTAGCACTAGAAAAACGAGAGGCT 225
P11CDO-S1_csep1C      TATTGGTGTTTAGATCAAGGTAATGATCTAGGCTGCCTAGCACTAGAAAAACGAGAGGCT 225
P7UCO-S2_csep1C       TATTGGTGTTTAGATCAAGGTAATGATCTAGGCTGCCTAGCACTAGAAAAACGAGAGGCT 225
H9O-S1_csep1C         TATTGGTGTTTAGATCAAGGTAATGATCTAGGCTGCCTAGCACTAGAAAAACGAGAGGCT 225
H20O-S1_csep1C        AGATGGTGCTTTGATAAAGGTAACGATCTAGGTTGTCTTTGGCTAGAAAAATTAGAGGCT 228
P18CDO-S1_csep1C      TATTGGTGCTTAGATCAAGGCAACGACCTAGGTTGTTTAGCATTAGAAAAATTAGAAGCT 225
H9O-S2_csep1C         TATTGGTGCTTAGATCAAGGTAACGATCTAGGTTGTTTAGCATTAGAAAAATTAGAAGCA 228
P13UCO-S1_csep1C      TATTGGTGCTTAGATCAAGGTAACGATCTAGGTTGTTTAGCATTAGAAAAATTAGAAGCA 225
P27CDO-S1_csep1C      TATTGGTGCTTAGATCAAGGTAACGATCTAGGTTGTTTAGCATTAGAAAAATTAGAAGCA 225
H29O-S1_csep1C        TATTGGTGCTTAGATCAAGGTAACGATCTAGGTTGTTTAGCATTAGAAAAATTAGAAGCA 225
P2CDO3_csep1C         TATTGGTGCCTTGATCAAGGCAATGATCTAGGTTGTATTTGGCTAGACAAACTAGAAGCT 234
P2CDO-S6_csep1C       TATTGGTGCCTTGATCAAGGCAATGATCTAGGTTGTATTTGGCTAGACAAACTAGAAGCT 234
P1CDO3_csep1C         TATTGGTGCCTTGATCAAGGCAATGATCTAGGTTGTATTTGGCTAGACAAACTAGAAGCT 237
P20CDO-S1_csep1C      TATTGGTGCCTTGATCAAGGCAATGATCTAGGTTGTATTTGGCTAGACAAACTAGAAGCT 234
P20CDO-S3_csep1C      TATTGGTGCCTTGATCAAGGCAATGATCTAGGTTGTATTTGGCTAGACAAACTAGAAGCT 234
H301_csep1C           TATTGGTGCCTTGATCAGGGCAATGATCTGGGTTGCATTTGGCTAGACAAACTAGAAGCT 237
P11CDO-S1_csep1C2     TATTGGTGTTTAGATCAAGGTAATGATCTAGGTTGTATTTGGCTAGATAAGCTAGAGGCC 237
P21CDO-S2_csep1C      TATTGGTGCCTTGATCAAGGCAATGATCTAGGTTGTATTTGGCTAGACAAACTAGAAGCT 234
H21O-S1_csep1C        TATTGGTGCCTTGATCAAGGCAATGATCTAGGTTGTATTTGGCTAGACAAACTAGAAGCT 237
                         *****  * **  * ** ** **  * ** **   *    **** **   *** **
```

Supplementary Figure S3

```
P2CDO4_csep1P          TTAGAGAGTAAAGACCTTAAAAAGGTTGTAAAATTCTACGAAAAAACTATCCCAGAATAC 288
P13UCO-S3_csep1P       TTAGAGAGCAAAGACCTTAAAAAGGTTGTAAAATTCTATGAAAAAACCATTCCAGAATAC 288
P26UCO-S1_csep1C       TTAGAGAGTAAAGACCTTAAAAAAGTTGTAAAATTCTACGAAAAAACCATTCCAGAATAC 285
P2CDO4_csep1C          TTAGAGAGTAAAGACCTTAAAAAGGTTATAAAATTCTACGAAAAAACCATTCCAGAATAC 285
P20CDO-S2_csep1P       TTAGAGAGTAAAGACCTTAAAAAGGTTATAAAATTCTACGAAAAAACCATTCCAGAATAC 285
P20CDO-S3_csep1P       TTAGAGAGTAAAGACCTTAAAAAGGTTATAAAATTCTACGAAAAAACCATTCCAGAATAC 285
P16UCO-S2_csep1C       ATGGAAAGCAAAGATCTTAAAAAAGTTGTAAATTTTTATGAGAAGACTATCCCAGAGTAT 288
H11O-S2_csep1C         TTAGCCAATAAAGACCTTAAAAAAGTAGTGAATTTTTATGAAAAAACTATTCCAGAATAC 285
P11CDO-S1_csep1C       TTAGCCAATAAAGACCTTAAAAAAGTAGTGAATTTTTATGAAAAAACTATTCCAGAATAC 285
P7UCO-S2_csep1C        TTAGCCAATAAAGACCTTAAAAAAGTAGTGAATTTTTATGAAAAAACAGTGCCAGAATAC 285
H9O-S1_csep1C          TTAGCCAATAAAGACCTTAAAAAAGTAGTGAATTTTTATGAAAAAACAGTGCCAGAATAC 285
H20O-S1_csep1C         ATGGAAAGCAAAGATCTTAAAAAAGTTGTAAATTTTTATGAGAAGACTATTCCAGAGTAT 288
P18CDO-S1_csep1C       TTAAAGGGTAAAGATCTCAAAAAAGTTATAAATTATTATGAAAAAACTATTCCAGAGTAT 285
H9O-S2_csep1C          TTAAAGAGTAAAGATCTTAAAAAGGTTATAAATTATTATGAAAAAACTATCCCTGAGTAT 288
P13UCO-S1_csep1C       TTAAAGAGTAAAGATCTTAAAAAGGTTATAAATTATTATGAAAAAACTATTCCAGAGTAT 285
P27CDO-S1_csep1C       TTAAAGAGTAAAGATCTTAAAAAGGTTATAAATTATTATGAAAAAACTATTCCAGAGTAT 285
H29O-S1_csep1C         TTAAAGAGTAAAGATCTTAAAAAGGTTATAAATTATTATGAAAAAACTATTCCAGAGTAT 285
P2CDO3_csep1C          TTAGCTAGTAAAGACATTAAGAAAGTAGCAAATTTTTATGAAAAAACAGTGCCAGAATAT 294
P2CDO-S6_csep1C        TTAGCTAGTAAAGACATTAAGAAAGTAGCAAATTTTTATGAAAAAACAGTGCCAGAATAT 294
P1CDO3_csep1C          TTAGCTAGTAAAGACATTAAGAAAGTAGCAAATTTTTATGAAAAAACAGTGCCAGAATAT 297
P20CDO-S1_csep1C       TTAGCTAGTAAAGACATTAAGAAAGTAGCAAATTTTTACGAAAAAACAGTGCCAGAATAT 294
P20CDO-S3_csep1C       TTAGCTAGTAAAGACATTAAGAAAGTAGCAAATTTTTACGAAAAAACAGTGCCAGAATAT 294
H3O1_csep1C            TTAGCTAGTAAAGACATTAAGAAAGTAACAAATTTTTATGAAAAAACAGTACCAGAATAT 297
P11CDO-S1_csep1C2      TTAGCTAGTAAAGACATTAAGAAAGTAGCAAATTTCTATGAAAAAACAGTGCCAGAATAT 297
P21CDO-S2_csep1C       TTAGCTAGTAAAGACATTAAGAAAGTAACAAATTTTTATGAAAAAACAGTGCCAGAATAT 294
H21O-S1_csep1C         TTAGCTAGTAAAGACATTAAGAAAGTAGCAAATTTTTATGAAAAAACAGTGCCAGAATAT 297
                        *       *****  * ** ** **    ** *  ** ** ** **  * ** ** **

P2CDO4_csep1P          TGCTACGATAAAAAAATTTGCTCCAGCTTGCAATATACCAGCCATTGATTTAATTCAACAA 348
P13UCO-S3_csep1P       TGCTACGATAAAAAAATTTGCTCCAGCGTGCAATATACCAGCCATTGATTTAATTCAACAA 348
P26UCO-S1_csep1C       TGCTACGATAAAAAAATTTGCTCCAGCATGCAATATACCAGCTATTGATTTAATTAAATAT 345
P2CDO4_csep1C          TGCTACGATAAAAAAATTTGCTCCAGCGTGCAATATACCAGCTATTGATTTAATTAAATAT 345
P20CDO-S2_csep1P       TGCTACGATAAAAAAATTTGCTCCAGCGTGCAATATACCAGCTATTGATTTAATTAAATAT 345
P20CDO-S3_csep1P       TGCTACGATAAAAAAATTTGCTCCAGCGTGCAATATACCAGCTATTGATTTAATTAAATAT 345
P16UCO-S2_csep1C       TGTTATGATAAAAAAATTTGCTCCAGCTTGCAATATACCAGCTCTTGATTTGATTAAAGAA 348
H11O-S2_csep1C         TGCTATGAGAAAAAAATTTGCTCCAGCGTGCAATATACCAGCTCTTGATTTGATTTTATAC 345
P11CDO-S1_csep1C       TGCTATGAGAAAAAAATTTGCTCCAGCGTGCAATATACCAGCTCTTGATTTGATTTTATAC 345
P7UCO-S2_csep1C        TGCTATGAGAAAAAAATTTGCTCCAGCGTGCAATATACCAGCTCTTGATTTGATTTTATAC 345
H9O-S1_csep1C          TGCTATGAGAAAAAAATTTGCTCCAGCGTGCAATATACCAGCTCTTGATTTGATTTTATAC 345
H20O-S1_csep1C         TGTTATGATAAAAAAATTTGCCCCAGCTTGCAATATACCAGCTCTTGATTTGATAAAATGG 348
P18CDO-S1_csep1C       TGTTATGATAAAAAAATTTGCTCCAGCATGCAATATACCAGCTCATGATTTGATAGAATGG 345
H9O-S2_csep1C          TGTTATGATAAAAAAATTTGCTCCAGCATGCAATATACCTGCTCTTGATTTGATAAAATGG 348
P13UCO-S1_csep1C       TGTTATGATAAAAAAATTTGCTCCAGCATGCAATATACCAGCTCTTGATTTGATAAAATGG 345
P27CDO-S1_csep1C       TGTTATGATAAAAAAATTTGCTCCAGCATGCAATATACCAGCTCTTGATTTGATAAAATGG 345
H29O-S1_csep1C         TGTTATGATAAAAAAATTTGCTCCAGCATGCAATATACCAGCTCTTGATTTGATAAAATGG 345
P2CDO3_csep1C          TGTTATGACAAAAAAATTTGCTCCAGCATGCAATATACCTGCTCTTGATTTGATAAAATGG 354
P2CDO-S6_csep1C        TGTTATGACAAAAAAATTTGCTCCAGCATGCAATATACCTGCTCTTGATTTGATAAAATGG 354
P1CDO3_csep1C          TGTTATGACAAAAAAATTTGCTCCAGCATGCAATATACCAGCTCGTGATTTGATAAAATGG 357
P20CDO-S1_csep1C       TGTTATGACAAAAAAATTTGCTCCAGCATGCAATATACCTGCTCTTGATTTGATAAAATGG 354
P20CDO-S3_csep1C       TGTTATGACAAAAAAATTTGCTCCAGCATGCAATATACCTGCTCTTGATTTGATAAAATGG 354
H3O1_csep1C            TGTTATGACAAAAAAATTCGCTCCAGCTTGCAATATACCAGCTCTTGATTTGATAAAATGG 357
P11CDO-S1_csep1C2      TGTTATGATAAAAAAATTCGCTCCAGCTTGCAGAATACCAGCATTTGATTTAATCAAAGAT 357
P21CDO-S2_csep1C       TGTTATGATAAAAAAATTCGCTCCAGCTTGCAATATACCAGCTCATGATTTGATAGATTGG 354
H21O-S1_csep1C         TGTTATGACAAAAAAATTCGCTCCAGCTTGCAATATACCAGCTCGTGATTTGATAGATTGG 357
                       ** ** ** ******** ** ***** ****  ***** **   ****** **
```

Supplementary Figure S3

```
                                                D
P2CDO4_csep1P         AAATTATCATACTATGTTAGAAATGATATTGATAATAAAACTATAAAAACTTTTTATTCT 408
P13UCO-S3_csep1P      AAATTATCATACTATGTTAGAAATGATATTGATAATAAAACTATAAAAACTTTTTATTCT 408
P26UCO-S1_csep1C      AAAATAGTTTATTTTTCAAAAAGAAGTGATGATTACACAAAAATAAAAAATCAAGAATTT 405
P2CDO4_csep1C         AAAATAGTTTATTTTTCAAAAAGAAGTGATGATTACACAAAAATAAAAAATCAAGAATTT 405
P20CDO-S2_csep1P      AAAATAGTTTATTTTTCAAAAAGAAGTGATGATTACACAAAAATAAAAAATCAAGAATTT 405
P20CDO-S3_csep1P      AAAATAGTTTATTTTTCAAAAAGAAGTGATGATTACACAAAAATAAAAAATCAAGAATTT 405
P16UCO-S2_csep1C      AAGGTAGTTTATTTTGCAAAGAAAAGTGGTGATTATGCAAAAGTAGAAGCCATAGATTCT 408
H11O-S2_csep1C        AAAATAATCTATTTTGCACATAAAAGCGATGATCAAGCTAAAACCGATGCCATAGATTCT 405
P11CDO-S1_csep1C      AAAATAATCTATTTTGCACATAAAAGCGATGATCAAGCTAAAACCGATGCCATAGATTCT 405
P7UCO-S2_csep1C       AAAATAATCTATTTTGCACATAAAAGCGATGATCAAGCTAAAACCGATGCCATAGATTCT 405
H9O-S1_csep1C         AAAATAATCTATTTTGCACATAAAAGCGATGATCAAGCTAAAACCGATGCCATAGATTCT 405
H20O-S1_csep1C        AAAATAGTTTATTTTGCAAAAAAAG---------ATAAAAATACCAAATCTATTTATTCT 399
P18CDO-S1_csep1C      AAAATAGTTTATTTTGCAAAAAAAAGTGATGATTATGCAAAAGAAAAAGCCATCGAGTCT 405
H9O-S2_csep1C         AAAATAGTTTATTTTGCAAAGAAAG---------ATGGAAATACTAAATCTATTTATTCT 399
P13UCO-S1_csep1C      AAAATAGTTTATTTTGCGAAGAAAG---------ATGGAAATACTAAATCTATTTATTCT 396
P27CDO-S1_csep1C      AAAATAGTTTATTTTGCGAAGAAAG---------ATGGAAATACTAAATCTATTTATTCT 396
H29O-S1_csep1C        AAAATAGTTTATTTTGCGAAGAAAG---------ATGGAAATACTAAATCTATTTATTCT 396
P2CDO3_csep1C         AAAATAGTTTATTTTGCAAAAAAAG---------ATAAAAATACCAAATCTATTTATTCT 405
P2CDO-S6_csep1C       AAAATAGTTTATTTTGCAAAAAAAG---------ATAAAAATACCAAATCTATTTATTCT 405
P1CDO3_csep1C         AAAATAGTTTATTTTGCAAAGAAAG---------ATGGAGATACTAAATCTATTTATTCT 408
P20CDO-S1_csep1C      AAAATAGTTTATTTTGCAAAAAAAG---------ATAAAAATACCAAATCTATTTATTCT 405
P20CDO-S3_csep1C      AAAATAGTTTATTTTGCAAAAAAAG---------ATAAAAATACCAAATCTATTTATTCT 405
H3O1_csep1C           AAAATAGTTTATTTTGCAAAGAAAG---------ATGGAGATACTAAATCTATTTATTCT 408
P11CDO-S1_csep1C2     AAAATTATATATTTTGCTAAAAAAA---------ATCAAAATACTAGCACTATAAACGCA 408
P21CDO-S2_csep1C      AAAATAGTTTATTTTGCAAAAAAAG---------ATAAAAATACCAAATCTATTTATTCT 405
H21O-S1_csep1C        AAAATAGTTTATTTTGCAAAAAAAG---------ATAAAAATACCAAATCTATTTATTCT 408
                      **  *    ** * *      *            *                    *

                                              E
P2CDO4_csep1P         GATTATGCAAAAGCCCTTTCTG---AGTCAAAAGCAGATGTAAAAATGCTTGAATATGGC 465
P13UCO-S3_csep1P      GACTATGCAAAAGCACTTTCTG---AGTCAAAAGCAGATGTAAAAATGCTTGAATATGGT 465
P26UCO-S1_csep1C      GATTATGCAAAAGTCCTTTCTG---AGTCAAAAGCAGACGTAAAAATGCTTGAATATGGC 462
P2CDO4_csep1C         GATTATGCAAAAGCCCTTTCTG---AGTCAAAAGCAGATGTAAAAATGCTTGAATATGGC 462
P20CDO-S2_csep1P      GATTATGCAAAAGCCCTTTCTG---AGTCAAAAGCAGACGTAAAAATGCTTGAATATGGC 462
P20CDO-S3_csep1P      GATTATGCAAAAGCCCTTTCTG---AGTCAAAAGCAGACGTAAAAATGCTTGAATATGGC 462
P16UCO-S2_csep1C      GATTATGCAAAAGCTCTTTCTG---AGTCAAAAGCGGACGTAAAAATGCTTGAATACGGC 465
H11O-S2_csep1C        GATTATGCAAAAGCTCTTTCTG---AGTCAAAAGCGGACGTAAAAATGCTTGAATACGGC 462
P11CDO-S1_csep1C      GATTACGCAAAAGCTCTTTCTG---AGTCAAAAGCGGACGTAAAAATGCTTGAATACGGC 462
P7UCO-S2_csep1C       GATTATGCAAAAGCTCTTTCTG---AGTCAAAAGCGGACGTAAAAATGCTTGAATACGGC 462
H9O-S1_csep1C         GATTATGCAAAAGCTCTTTCTG---AGTCAAAAGCGGACGTAAAAATGCTTGAATACGGC 462
H20O-S1_csep1C        GACTATGCAAAAGCACTTTCTG---AGTCAAAAGCAGATGTAAAAATGCTTGAATATGGT 456
P18CDO-S1_csep1C      GACTATGCAAAAGCACTTTCTG---AGTCAAAAGCAGATGTAAAAATGCTTGAATATGGC 462
H9O-S2_csep1C         GACTATGCAAAAGCACTTTCTG---AGTCAAAAGCAGATGTAAAAATGCTTGAATATGGC 456
P13UCO-S1_csep1C      GACTATGCAAAAGCACTTTCTG---AGTCAAAAGCAGATGTAAAAATGCTTGAATATGGT 453
P27CDO-S1_csep1C      GACTATGCAAAAGCACTTTCTG---AGTCAAAAGCAGATGTAAAAATGCTTGAATATGGT 453
H29O-S1_csep1C        GACTATGCAAAAGCACTTTCTG---AGTCAAAAGCAGATGTAAAAATGCTTGAATATGGT 453
P2CDO3_csep1C         GACTATGCAAAAGCACTTTCTG---AGTCAAAAGCAGATGTAAAAATGCTTGAATATGGC 462
P2CDO-S6_csep1C       GACTATGCAAAAGCACTTTCTG---AGTCAAAAGCAGATGTAAAAATGCTTGAATATGGC 462
P1CDO3_csep1C         GACTATGCAAAAGCACTTTCTG---AGTCAAAAGCAGATGTAAAAATGCTTGAATATGGC 465
P20CDO-S1_csep1C      GACTATGCAAAAGCACTTTCTG---AGTCAAAAGCAGATGTAAAAATGCTTGAATATGGC 462
P20CDO-S3_csep1C      GACTATGCAAAAGCACTTTCTG---AGTCAAAAGCAGATGTAAAAATGCTTGAATATGGC 462
H3O1_csep1C           GACTATGCAAAAGCACTTTCTG---AGTCAAAAGCAGATGTAAAAATGCTTGAATATGGT 465
P11CDO-S1_csep1C2     GATTTTGCAAAAATGATTACTGCCAAATCAGGAGCAGAGCTTAAAATGCTTGAGTATGGT 468
P21CDO-S2_csep1C      GACTATGCAAAAGCACTTTCTG---AGTCAAAAGCGGACGTAAAAATGCTTGAATATGGC 462
H21O-S1_csep1C        GACTATGCAAAAGCACTTTCTG---AGTCAAAAGCAGATGTAAAAATGCTTGAATATGGC 465
                      ** *  ******    ** ***   * ***  *** **  * *********** ** **
```

Supplementary Figure S3

```
P2CDO4_csep1P           TGCAATGAATTAAAAAGTGCCTATATATGTAGAGATCTGCGCGATATGTATAAATACTTA 525
P13UCO-S3_csep1P        TGCAATGAACTAAAAAGTGCCTATATATGTAGGGATCTACGTGACATGTATAAAAACTTA 525
P26UCO-S1_csep1C        TGCAATGAACTAAAAAGTGCCTATATATGTAGAGATCTACGCGATATGTATAAATATTTA 522
P2CDO4_csep1C           TGCAATGAACTAAAAAGTGCCTATATATGTAGAGATCTACGCGACATGTATAAGTATTTA 522
P20CDO-S2_csep1P        TGCAATGAACTAAAAAGTGCCTATATATGTAGAGATCTACGCGATATGTATAAATATTTA 522
P20CDO-S3_csep1P        TGCAATGAACTAAAAAGTGCCTATATATGTAGAGATCTACGCGATATGTATAAATATTTA 522
P16UCO-S2_csep1C        TGCAATGAGCTAAAAAGCGCATATATATGTAGAGATCTACGCGACATGTATAAAAACCTG 525
H11O-S2_csep1C          TGCAATGAGCTAAAAAGCGCATATATATGTAGAGATCTACGCGACATGTATAAAAACCTG 522
P11CDO-S1_csep1C        TGCAATGAGCTAAAAAGCGCATATATATGTAGAGATCTACGCGACATGTATAAAAACCTG 522
P7UCO-S2_csep1C         TGCAATGAGCTAAAAAGCGCATATATATGTAGAGATCTACGCGACATGTATAAAAACCTG 522
H9O-S1_csep1C           TGCAATGAGCTAAAAAGCGCATATATATGTAGAGATCTACGCGACATGTATAAAAACCTG 522
H20O-S1_csep1C          TGTAATGAACTAAAAAGTTCCTATATATGTAGAGATCTGCGTGATATGTATAAAAACTTA 516
P18CDO-S1_csep1C        TGCAATGAGTTAAAAAGTGCCTATATATGTAGAGATCTACGTGACATGTATAAAATTTTA 522
H9O-S2_csep1C           TGCAATGAATTAAAAAGTGCCTATATATGTAGAGATCTACGTGACATGTATAAAAATTTA 516
P13UCO-S1_csep1C        TGTAATGAACTAAAAAGTGCCTATATATGTAGAGATCTACGTGACATGTATAAAAATTTA 513
P27CDO-S1_csep1C        TGTAATGAACTAAAAAGTGCCTATATATGTAGAGATCTACGTGACATGTATAAAAATTTA 513
H29O-S1_csep1C          TGTAATGAACTAAAAAGTGCCTATATATGTAGAGATCTACGTGACATGTATAAAAATTTA 513
P2CDO3_csep1C           TGCAATGAACTAAAAAGCGCCTATATATGTAGAGATCTACGTGACATGTATAAAAATTTA 522
P2CDO-S6_csep1C         TGCAATGAACTAAAAAGCGCCTATATATGTAGAGATCTACGTGACATGTATAAAAATTTA 522
P1CDO3_csep1C           TGCAATGAACTAAAAAGTGCCTATATATGTAGAGATCTACGCGATATGTATAAATATTTA 525
P20CDO-S1_csep1C        TGCAATGAACTAAAAAGCGCCTATATATGTAGAGATCTACGTGACATGTATAAAAATTTA 522
P20CDO-S3_csep1C        TGCAATGAACTAAAAAGCGCCTATATATGTAGAGATCTACGTGACATGTATAAAAATTTA 522
H3O1_csep1C             TGCAATGAACTAAAAAGTGCCTATATATGTAGGGATCTACGTGACATGTATAAAAACTTA 525
P11CDO-S1_csep1C2       TGCAATGAGCTTAAAAGTGGATATATATGTAGGGATTTGCGCGAAATATATAAAAATCTC 528
P21CDO-S2_csep1C        TGCAATGAACTAAAAAGTGCCTATATATGTAGAGATCTACGCGATATGTATAAAAATTTA 522
H21O-S1_csep1C          TGCAATGAACTAAAAAGCGCCTATATATGTAGAGATCTACGTGACATGTATAAAAATTTA 525
                        ** *****  * *****    *********** *** * ** ** ** *****     *

P2CDO4_csep1P           GGCGACAAAGAAAAAACGAAAGAGTATAACGACAAAATGAAAAATGGCGATGAGAAGTGG 585
P13UCO-S3_csep1P        GGCGATAGAGAAAAGACTAAAGAATATAACGATAAAATGAAAAATGGTGATGAAAAGTGG 585
P26UCO-S1_csep1C        GGTAACAAAGAAAAGACAAAAGAGTATAACGACAAAATGAAAAATGGTGATGAGAAGTGG 582
P2CDO4_csep1C           GGCGATAGAAAAAAGACTAAAGAGTATAACGATAAGATGAAAAATGGTGATGAGAAGTGG 582
P20CDO-S2_csep1P        GGCGACAAAGAAAAGACAAAAGAGTATAATGACAAAATGAAAAATGGTGATGAGAAGTGG 582
P20CDO-S3_csep1P        GGCGACAAAGAAAAGACAAAAGAGTATAATGACAAAATGAAAAATGGTGATGAGAAGTGG 582
P16UCO-S2_csep1C        GGCGATAGAGATAAAATAAGAGAATATAACGAGAAAATAAAAAACGGTGATAAAAAATGG 585
H11O-S2_csep1C          GGCGATAGAGATAAAATAAGAGAATATAACGAGAAAATAAAAAACGGTGATAAAAAATGG 582
P11CDO-S1_csep1C        GGCGATAGAGATAAAATAAGAGAATATAACGAGAAAATAAAAAACGGTGATAAAAAATGG 582
P7UCO-S2_csep1C         GGCGATAGAGATAAAATAAGAGAATATAACGAGAAAATAAAAAACGGTGATAAAAAATGG 582
H9O-S1_csep1C           GGCGATAGAGATAAAATAAGAGAATATAACGAGAAAATAAAAAACGGTGATAAAAAATGG 582
H20O-S1_csep1C          GGCGATAGAGAAAAGACTAAAGAATATAACGACAAAATGAAAAGTGGTGATGAAAAGTGG 576
P18CDO-S1_csep1C        GGCGATAGAGAAAAGACAAAAGAATATAACGACAAAATGAAAAATGGTGATGAAAAATGG 582
H9O-S2_csep1C           GGCGACAGAGAAAAGACTAAAGAGTATAACGACAAAATGAAAAATGGTGATGAAAAATGG 576
P13UCO-S1_csep1C        GGCGATAGAGAAAAGACAAAAGAATATAACGATAAAATGAAAAATGGCAATGAAAAATGG 573
P27CDO-S1_csep1C        GGCGATAGAGAAAAGACAAAAGAATATAACGATAAAATGAAAAATGGCAATGAAAAATGG 573
H29O-S1_csep1C          GGCGATAGAGAAAAGACAAAAGAATATAACGATAAAATGAAAAATGGCAATGAAAAATGG 573
P2CDO3_csep1C           GGCGATAGAGAAAAGACAAAAGAGTATAACGACAAAATGAAAAATGGTGATGAAAAGTGG 582
P2CDO-S6_csep1C         GGCGATAGAGAAAAGACAAAAGAGTATAACGACAAAATGAAAAATGGTGATGAAAAGTGG 582
P1CDO3_csep1C           GGCGACAAAGAAAAGACAAAAGAGTATAACGACAAAATGAAAAATGGTGATGAGAAGTGG 585
P20CDO-S1_csep1C        GGCGATAGAGAAAAGACTAAAGAGTATAACGACAAAATGAAAAATGGTGATGAAAAGTGG 582
P20CDO-S3_csep1C        GGCGATAGAGAAAAGACTAAAGAGTATAACGACAAAATGAAAAATGGTGATGAAAAGTGG 582
H3O1_csep1C             GGCGATAGAGAAAAGACTAAAGAATATAACGATAAAATGAAAAATGGTGATGAAAAGTGG 585
P11CDO-S1_csep1C2       GATGATAGGGAAAAAATAAAAGAATATAACGATAAGATAAAAAATAGAGATAAAAAATGG 588
P21CDO-S2_csep1C        GGCGACATAGAAAAGACTAAAGAGTATAACGACAAAATGAAAAATGGTGATGAGAAGTGG 582
H21O-S1_csep1C          GGCGATAGAGAAAAGACAAAAGAATATAACGACAAAATGAAAAATGGCGATGAAAAGTGG 585
                        *   * *   * ** *  * *** ***** ** ** ** ****  *   ** * ** ***
```

Supplementary Figure S3

```
P2CDO4_csep1P         AATTCAGTTCTTTATGACTATAAGCACATGCGATATATCCACGGTGGTTATTCAAGCTGG 645
P13UCO-S3_csep1P      AATTCTGTTCTTTATGACTATCAACACATGAGATATATTTATAAAGATGCCTCTGCGTGG 645
P26UCO-S1_csep1C      AATTCGGTTCTTTATGATTATAAGCACATGCGATATATCCACGGTGGTTATTCAAGCTGG 642
P2CDO4_csep1C         AATTCGGTTCTTTATGATTATAAGCACATGCGATATATCCACGGTGGTTATTCAAGCTGG 642
P20CDO-S2_csep1P      AATTCGGTTCTTTATGATTATAAGCACATGCGATATATCCACGGTGGTTATTCAAGCTGG 642
P20CDO-S3_csep1P      AATTCGGTTCTTTATGATTATAAGCACATGCGATATATCCACGGTGGTTATTCAAGCTGG 642
P16UCO-S2_csep1C      AATTCAGTCATTTATGATTATCATCATAGTCGATTTGCCTATGGAAGAGCTTCTGGATGG 645
H11O-S2_csep1C        AATTCAGTCATTTATGATTATCATCATAGTCGATTTGCCTATGGAAGAGCTTCTGGATGG 642
P11CDO-S1_csep1C      AATTCAGTCATTTATGATTATCATCATAGTCGATTTGCCTATGGAAGAGCTTCTGGATGG 642
P7UCO-S2_csep1C       AATTCAGTCATTTATGATTATCATCATAGTCGATTTGCCTATGGAAGAGCTTCTGGATGG 642
H9O-S1_csep1C         AATTCAGTCATTTATGATTATCATCATAGTCGATTTGCCTATGGAAGAGCTTCTGGATGG 642
H20O-S1_csep1C        AATTCGGTCCTTTATGATTATCAGCACATGAGATATATAAATAGTAATAACTCTGCATGG 636
P18CDO-S1_csep1C      AATTCTGTTCTTTATGACTATCAGCACATGAGATATATAAATAGCAATAACTCTGCATGG 642
H9O-S2_csep1C         AATTCAGTTCTTTACGATTATCAACATAGACGATACATAAATAGGAATAACTCTGCATGG 636
P13UCO-S1_csep1C      AATTCTGTTCTTTACGATTATCAACATAGACGATACATAAATAGGAATAACTCTGCATGG 633
P27CDO-S1_csep1C      AATTCTGTTCTTTACGATTATCAACATAGACGATACATAAATAGGAATAACTCTGCATGG 633
H29O-S1_csep1C        AATTCTGTTCTTTACGATTATCAACATAGACGATACATAAATAGGAATAACTCTGCATGG 633
P2CDO3_csep1C         AATTCTGTTCTTTATGATTATAAACACATGCGATATATCCACGGTGATTATTCAAGCTGG 642
P2CDO-S6_csep1C       AATTCTGTTCTTTATGATTATAAACACATGCGATATATCCACGGTGATTATTCAAGCTGG 642
P1CDO3_csep1C         GATTCGGTTCTTTATGATTATAAGCACATGCGATATATCCACGGTGGTTATTCAAGCTGC 645
P20CDO-S1_csep1C      AATTCAGTTCTTTATGATTATCAACACATGAGATATATCTATAAAGATGCTTCTGCATGG 642
P20CDO-S3_csep1C      AATTCAGTTCTTTATGATTATCAACACATGAGATATATCTATAAAGATGCTTCTGCATGG 642
H3O1_csep1C           AATTCTGTTCTTTATGACTATCAACACATGAGATATATTTATAAAGATGCCTCTGCGTGG 645
P11CDO-S1_csep1C2     GACTCTGTGTTATATGATAGTAATCACATGAGATATATATATAAAAATGCGTCAGGATGG 648
P21CDO-S2_csep1C      AATTCAGTTCTTTATGATTATCAACACATGAGATATATTTATAAAGACGCCACTGCGTGG 642
H21O-S1_csep1C        AATTCTGTTCTTTATGACTATCAACACATAAGATATATTTATAAAGACGCCTCTGCGTGG 645
                       * ** **  * ** **   * * ** *   ***      *           *    **

                              F
P2CDO4_csep1P         TGGCTTTTAGAAAAAATAAAATAA 669
P13UCO-S3_csep1P      AAGCTTTT------AATCAATTAA 663
P26UCO-S1_csep1C      TTGCTTTTAGAAAAGATAAAGTAA 666
P2CDO4_csep1C         TTGCTTTTAGAAAAGATAAAGTAA 666
P20CDO-S2_csep1P      TTGCTTTTAGAAAAAATAAAATAG 666
P20CDO-S3_csep1P      TTGCTTTTAGAAAAAATAAAATAG 666
P16UCO-S2_csep1C      TGGTTTTT------GGACGATTGA 663
H11O-S2_csep1C        TGGTTTTT------GGACGATTGA 660
P11CDO-S1_csep1C      TGGTTTTT------GGACGATTGA 660
P7UCO-S2_csep1C       TGGTTTTT------GGACGATTGA 660
H9O-S1_csep1C         TGGTTTTT------GGACGATTGA 660
H20O-S1_csep1C        TGGCTTTT------AGAAGATTAG 654
P18CDO-S1_csep1C      TGGCTTTT------GCTCTATTAA 660
H9O-S2_csep1C         TGGCTTTT------AGAAGATTAA 654
P13UCO-S1_csep1C      TGGCTTTT------GCTCTATTAA 651
P27CDO-S1_csep1C      TGGCTTTT------GCTCTATTAA 651
H29O-S1_csep1C        TGGCTTTT------GCTCTATTAA 651
P2CDO3_csep1C         TGGCTTTTAGAAAAAATAAAGTAA 666
P2CDO-S6_csep1C       TGGCTTTTAGAAAAAATAAAGTAA 666
P1CDO3_csep1C         TTGCTTTTAGAAAAAATAAAGTAA 669
P20CDO-S1_csep1C      TGGCTTTT------GCTCTATTAA 660
P20CDO-S3_csep1C      TGGCTTTT------GCTCTATTAA 660
H3O1_csep1C           AAGCTTTT------AATCAATTAA 663
P11CDO-S1_csep1C2     AGGAATTTAGAGTTTCTTAAATAG 672
P21CDO-S2_csep1C      AAGCTTTT------GCTCTATTAA 660
H21O-S1_csep1C        AAGCTTTT------AATCAATTAA 663
                        *  ***           * *
```
