# Supplementary material for: Genomic analysis of oral Campylobacter concisus strains identified a potential bacterial molecular marker associated with active Crohn’s disease
Source: Emerg Microbes Infect. 2018 Apr 11;7:64. doi: 10.1038/s41426-018-0065-6 (PMC5893538; doi:10.1038/s41426-018-0065-6)
Supplement: Supplementary file 6 — Supplementary Figure S4 [file 41426_2018_65_MOESM6_ESM.pdf]

Supplementary Figure S4

```
P2CDO4_Csep1P        MKKQILASVLASVLATSSVYAWGFGGSSKEKPNYDYSADKKLIEINT---RPISTDNAKY 57
P13UCO-S3_Csep1C     MKKQILASVLASVLATSSVYAWGFGGSSKEKPNYDYSADKKLIEINT---RPISTDNAKY 57
P26UCO-S1_Csep1C     MKKQILASVLASVLATSSVYAWGF-GSSREKPNYDYSADKKLIEINT---RPISTDNAKY 56
P2CDO4_Csep1C        MKKQILASVLASVLAISSVYAWGF-GSSREKPNYDYSGEKEFIQKNT---KEIGGDNAKY 56
P20CDO-S2_Csep1P     MKKQILASVLASVLATSSVYAWGF-GSSKEKPNYDYSGEKEFIQKNT---KEIGGDNAKY 56
P20CDO-S3_Csep1P     MKKQILASVLASVLATSSVYAWGF-GSSKEKPNYDYSGEKEFIQKNT---KEIGGDNAKY 56
P16UCO-S2_Csep1C     MKKQILASVLVGALAVSSASAWSLFGDDREKPNYDYSGEKEFIQKNT---QKLGTNDAKR 57
H11O-S2_Csep1C       MKKQILASVLASVLATSSVYAWGF-GSSKEKPNYDYSGDKQFIETNT---KKIDTDRAKY 56
P11CDO-S1_Csep1C     MKKQILASVLASVLATSSVYAWGF-GSSKEKPNYDYSGDKQFIETNT---KKIDTDRAKY 56
P7UCO-S2_Csep1C      MKKQILASVLASALATSSVYAWGF-GSSKEKPNYDYSGDKQFIETNT---KKIDTDRAKY 56
H9O-S1_Csep1C        MKKQILASVLASALATSSVYAWGF-GSSKEKPNYDYSGDKQFIETNT---KKIDTDRAKY 56
H20O-S1_Csep1C       MKKQILASVLASVLATSSVYAWGFGGSSKEKPNYDYSGEKEFIQKNT---QKLGTNDAKR 57
P18CDO-S1_Csep1C     MKKQILASVLASVLATSSVYAWGF-GSSREKPNYDYSADKKLIETNT---QKIGTEKAKY 56
H9O-S2_Csep1C        MKKQILASILVGALAVSSASAWSLFGGEREKPNYDYSADKKLIETNT---QKIGTEKAKY 57
P13UCO-S1_Csep1C     MKKQILASVLASVLATSSVYAWGF-GSSREKPNYDYSADKKLIETNT---QKIGTEKAKY 56
P27CDO-S1_Csep1C     MKKQILASVLASVLATSSVYAWGF-GSSREKPNYDYSADKKLIETNT---QKIGTEKAKY 56
H29O-S1_Csep1C       MKKQILASVLASVLATSSVYAWGF-GSSREKPNYDYSADKKLIETNT---QKIGTEKAKY 56
P2CDO3_Csep1C        MKKQILASVLASVLATSSVYAWGF-GSSKEKPNYDYSGEKELMSLSSFSDKDLRIDDAKY 59
P2CDO-S6_Csep1C      MKKQILASVLASVLATSSVYAWGF-GSSKEKPNYDYSGEKELMSLSSFSDKDLRIDDAKY 59
P1CDO3_Csep1C        MKKQILASVLVGALTAGSASAWSLFGGDREKPNYDYSGEKELMSLSSFSDKDLRIDDAKY 60
P20CDO-S1_Csep1C     MKKQILASILASVLATSSVYAWGF-GSSREKPNYDYSGEKELMSLSSFSDKDLRIDDAKY 59
P20CDO-S3_Csep1C     MKKQILASILASVLATSSVYAWGF-GSSREKPNYDYSGEKELMSLSSFSDKDLRIDDAKY 59
H3O1_Csep1C          MKKQILASVLASVLATSSVYAWGFGGSSKEKPNYDYSGEKELMSLSSFSDKDLRIDDAKY 60
P11CDO-S1_Csep1C2    MKKQILASVLASVLATSSVYAWGFGGSSKEKPNYDYSGEKELMSLSSFSGNNLRIDDAKY 60
P21CDO-S2_Csep1C     MKKQILASVLASVLAISSVYAWGF-GSSREKPNYDYSGEKELMSLSSFSDKDLRIDDAKY 59
H21O-S1_Csep1C       MKKQILASVLASVLATSSVYAWGFGGSSREKPNYDYSGEKELMSLSSFSDKDLRIDDAKY 60
                     ********:*...*: .*. **.: *..:********.:*:::. .:   . :  : **

P2CDO4_Csep1P        WCFDKGNDLGCLSLEKLEALESKDLKKVVKFYEKTIPEYCYDKKFAPACNIPAIDLIQQK 117
P13UCO-S3_Csep1C     WCFDKGNDLGCLSLEKLEALESKDLKKVVKFYEKTIPEYCYDKKFAPACNIPAIDLIQQK 117
P26UCO-S1_Csep1C     WCFDKGNDLGCLSLEKLEALESKDLKKVVKFYEKTIPEYCYDKKFAPACNIPAIDLIKYK 116
P2CDO4_Csep1C        WCFDKGNDLGCLSLEKLEALESKDLKKVIKFYEKTIPEYCYDKKFAPACNIPAIDLIKYK 116
P20CDO-S2_Csep1P     WCFDKGNDLGCLSLEKLEALESKDLKKVIKFYEKTIPEYCYDKKFAPACNIPAIDLIKYK 116
P20CDO-S3_Csep1P     WCFDKGNDLGCLSLEKLEALESKDLKKVIKFYEKTIPEYCYDKKFAPACNIPAIDLIKYK 116
P16UCO-S2_Csep1C     WCFDKGNDLGCLWLEKLEAMESKDLKKVVNFYEKTIPEYCYDKKFAPACNIPALDLIKEK 117
H11O-S2_Csep1C       WCLDQGNDLGCLALEKREALANKDLKKVVNFYEKTIPEYCYEKKFAPACNIPALDLILYK 116
P11CDO-S1_Csep1C     WCLDQGNDLGCLALEKREALANKDLKKVVNFYEKTIPEYCYEKKFAPACNIPALDLILYK 116
P7UCO-S2_Csep1C      WCLDQGNDLGCLALEKREALANKDLKKVVNFYEKTVPEYCYEKKFAPACNIPALDLILYK 116
H9O-S1_Csep1C        WCLDQGNDLGCLALEKREALANKDLKKVVNFYEKTVPEYCYEKKFAPACNIPALDLILYK 116
H20O-S1_Csep1C       WCFDKGNDLGCLWLEKLEAMESKDLKKVVNFYEKTIPEYCYDKKFAPACNIPALDLIKWK 117
P18CDO-S1_Csep1C     WCLDQGNDLGCLALEKLEALKGKDLKKVINYYEKTIPEYCYDKKFAPACNIPAHDLIEWK 116
H9O-S2_Csep1C        WCLDQGNDLGCLALEKLEALKSKDLKKVINYYEKTIPEYCYDKKFAPACNIPALDLIKWK 117
P13UCO-S1_Csep1C     WCLDQGNDLGCLALEKLEALKSKDLKKVINYYEKTIPEYCYDKKFAPACNIPALDLIKWK 116
P27CDO-S1_Csep1C     WCLDQGNDLGCLALEKLEALKSKDLKKVINYYEKTIPEYCYDKKFAPACNIPALDLIKWK 116
H29O-S1_Csep1C       WCLDQGNDLGCLALEKLEALKSKDLKKVINYYEKTIPEYCYDKKFAPACNIPALDLIKWK 116
P2CDO3_Csep1C        WCLDQGNDLGCIWLDKLEALASKDIKKVANFYEKTVPEYCYDKKFAPACNIPALDLIKWK 119
P2CDO-S6_Csep1C      WCLDQGNDLGCIWLDKLEALASKDIKKVANFYEKTVPEYCYDKKFAPACNIPALDLIKWK 119
P1CDO3_Csep1C        WCLDQGNDLGCIWLDKLEALASKDIKKVANFYEKTVPEYCYDKKFAPACNIPARDLIKWK 120
P20CDO-S1_Csep1C     WCLDQGNDLGCIWLDKLEALASKDIKKVANFYEKTVPEYCYDKKFAPACNIPALDLIKWK 119
P20CDO-S3_Csep1C     WCLDQGNDLGCIWLDKLEALASKDIKKVANFYEKTVPEYCYDKKFAPACNIPALDLIKWK 119
H3O1_Csep1C          WCLDQGNDLGCIWLDKLEALASKDIKKVTNFYEKTVPEYCYDKKFAPACNIPALDLIKWK 120
P11CDO-S1_Csep1C2    WCLDQGNDLGCIWLDKLEALASKDIKKVANFYEKTVPEYCYDKKFAPACRIPAFDLIKDK 120
P21CDO-S2_Csep1C     WCLDQGNDLGCIWLDKLEALASKDIKKVTNFYEKTVPEYCYDKKFAPACNIPAHDLIDWK 119
H21O-S1_Csep1C       WCLDQGNDLGCIWLDKLEALASKDIKKVANFYEKTVPEYCYDKKFAPACNIPARDLIDWK 120
                     **:*:******: *:* **: .**:*** ::****:*****:*******.*** ***  *
```

Supplementary Figure S4

```
P2CDO4_Csep1P         LSYYVRNDIDNKTIKTFYSDYAKALS-ESKADVKMLEYGCNELKSAYICRDLRDMYKYLG 176
P13UCO-S3_Csep1C      LSYYVRNDIDNKTIKTFYSDYAKALS-ESKADVKMLEYGCNELKSAYICRDLRDMYKNLG 176
P26UCO-S1_Csep1C      IVYFSKRSDDYTKIKNQEFDYAKVLS-ESKADVKMLEYGCNELKSAYICRDLRDMYKYLG 175
P2CDO4_Csep1C         IVYFSKRSDDYTKIKNQEFDYAKALS-ESKADVKMLEYGCNELKSAYICRDLRDMYKYLG 175
P20CDO-S2_Csep1P      IVYFSKRSDDYTKIKNQEFDYAKALS-ESKADVKMLEYGCNELKSAYICRDLRDMYKYLG 175
P20CDO-S3_Csep1P      IVYFSKRSDDYTKIKNQEFDYAKALS-ESKADVKMLEYGCNELKSAYICRDLRDMYKYLG 175
P16UCO-S2_Csep1C      VVYFAKKSGDYAKVEAIDSDYAKALS-ESKADVKMLEYGCNELKSAYICRDLRDMYKNLG 176
H11O-S2_Csep1C        IIYFAHKSDDQAKTDAIDSDYAKALS-ESKADVKMLEYGCNELKSAYICRDLRDMYKNLG 175
P11CDO-S1_Csep1C      IIYFAHKSDDQAKTDAIDSDYAKALS-ESKADVKMLEYGCNELKSAYICRDLRDMYKNLG 175
P7UCO-S2_Csep1C       IIYFAHKSDDQAKTDAIDSDYAKALS-ESKADVKMLEYGCNELKSAYICRDLRDMYKNLG 175
H9O-S1_Csep1C         IIYFAHKSDDQAKTDAIDSDYAKALS-ESKADVKMLEYGCNELKSAYICRDLRDMYKNLG 175
H20O-S1_Csep1C        IVYFAKKDKN---TKSIYSDYAKALS-ESKADVKMLEYGCNELKSSYICRDLRDMYKNLG 173
P18CDO-S1_Csep1C      IVYFAKKSDDYAKEKAIESDYAKALS-ESKADVKMLEYGCNELKSAYICRDLRDMYKILG 175
H9O-S2_Csep1C         IVYFAKKDGN---TKSIYSDYAKALS-ESKADVKMLEYGCNELKSAYICRDLRDMYKNLG 173
P13UCO-S1_Csep1C      IVYFAKKDGN---TKSIYSDYAKALS-ESKADVKMLEYGCNELKSAYICRDLRDMYKNLG 172
P27CDO-S1_Csep1C      IVYFAKKDGN---TKSIYSDYAKALS-ESKADVKMLEYGCNELKSAYICRDLRDMYKNLG 172
H29O-S1_Csep1C        IVYFAKKDGN---TKSIYSDYAKALS-ESKADVKMLEYGCNELKSAYICRDLRDMYKNLG 172
P2CDO3_Csep1C         IVYFAKKDKN---TKSIYSDYAKALS-ESKADVKMLEYGCNELKSAYICRDLRDMYKNLG 175
P2CDO-S6_Csep1C       IVYFAKKDKN---TKSIYSDYAKALS-ESKADVKMLEYGCNELKSAYICRDLRDMYKNLG 175
P1CDO3_Csep1C         IVYFAKKDGD---TKSIYSDYAKALS-ESKADVKMLEYGCNELKSAYICRDLRDMYKYLG 176
P20CDO-S1_Csep1C      IVYFAKKDKN---TKSIYSDYAKALS-ESKADVKMLEYGCNELKSAYICRDLRDMYKNLG 175
P20CDO-S3_Csep1C      IVYFAKKDKN---TKSIYSDYAKALS-ESKADVKMLEYGCNELKSAYICRDLRDMYKNLG 175
H3O1_Csep1C           IVYFAKKDGD---TKSIYSDYAKALS-ESKADVKMLEYGCNELKSAYICRDLRDMYKNLG 176
P11CDO-S1_Csep1C2     IIYFAKKNQN---TSTINADFAKMITAKSGAELKMLEYGCNELKSGYICRDLREIYKNLD 177
P21CDO-S2_Csep1C      IVYFAKKDKN---TKSIYSDYAKALS-ESKADVKMLEYGCNELKSAYICRDLRDMYKNLG 175
H21O-S1_Csep1C        IVYFAKKDKN---TKSIYSDYAKALS-ESKADVKMLEYGCNELKSAYICRDLRDMYKNLG 176
                      : *: :.. :    .    *:** :: :* *::************.*******::** *.

P2CDO4_Csep1P         DKEKTKEYNDKMKNGDEKWNSVLYDYKHMRYIHGGYSSWWLLEKIK 222
P13UCO-S3_Csep1C      DREKTKEYNDKMKNGDEKWNSVLYDYQHMRYIYKDASAWKLL--IN 220
P26UCO-S1_Csep1C      NKEKTKEYNDKMKNGDEKWNSVLYDYKHMRYIHGGYSSWLLLEKIK 221
P2CDO4_Csep1C         DRKKTKEYNDKMKNGDEKWNSVLYDYKHMRYIHGGYSSWLLLEKIK 221
P20CDO-S2_Csep1P      DKEKTKEYNDKMKNGDEKWNSVLYDYKHMRYIHGGYSSWLLLEKIK 221
P20CDO-S3_Csep1P      DKEKTKEYNDKMKNGDEKWNSVLYDYKHMRYIHGGYSSWLLLEKIK 221
P16UCO-S2_Csep1C      DRDKIREYNEKIKNGDKKWNSVIYDYHHSRFAYGRASGWWFL--DD 220
H11O-S2_Csep1C        DRDKIREYNEKIKNGDKKWNSVIYDYHHSRFAYGRASGWWFL--DD 219
P11CDO-S1_Csep1C      DRDKIREYNEKIKNGDKKWNSVIYDYHHSRFAYGRASGWWFL--DD 219
P7UCO-S2_Csep1C       DRDKIREYNEKIKNGDKKWNSVIYDYHHSRFAYGRASGWWFL--DD 219
H9O-S1_Csep1C         DRDKIREYNEKIKNGDKKWNSVIYDYHHSRFAYGRASGWWFL--DD 219
H20O-S1_Csep1C        DREKTKEYNDKMKSGDEKWNSVLYDYQHMRYINSNNSAWWLL--ED 217
P18CDO-S1_Csep1C      DREKTKEYNDKMKNGDEKWNSVLYDYQHMRYINSNNSAWWLL--LY 219
H9O-S2_Csep1C         DREKTKEYNDKMKNGDEKWNSVLYDYQHRRYINRNNSAWWLL--ED 217
P13UCO-S1_Csep1C      DREKTKEYNDKMKNGNEKWNSVLYDYQHRRYINRNNSAWWLL--LY 216
P27CDO-S1_Csep1C      DREKTKEYNDKMKNGNEKWNSVLYDYQHRRYINRNNSAWWLL--LY 216
H29O-S1_Csep1C        DREKTKEYNDKMKNGNEKWNSVLYDYQHRRYINRNNSAWWLL--LY 216
P2CDO3_Csep1C         DREKTKEYNDKMKNGDEKWNSVLYDYKHMRYIHGDYSSWWLLEKIK 221
P2CDO-S6_Csep1C       DREKTKEYNDKMKNGDEKWNSVLYDYKHMRYIHGDYSSWWLLEKIK 221
P1CDO3_Csep1C         DKEKTKEYNDKMKNGDEKWDSVLYDYKHMRYIHGGYSSCLLLEKIK 222
P20CDO-S1_Csep1C      DREKTKEYNDKMKNGDEKWNSVLYDYQHMRYIYKDASAWWLL--LY 219
P20CDO-S3_Csep1C      DREKTKEYNDKMKNGDEKWNSVLYDYQHMRYIYKDASAWWLL--LY 219
H3O1_Csep1C           DREKTKEYNDKMKNGDEKWNSVLYDYQHMRYIYKDASAWKLL--IN 220
P11CDO-S1_Csep1C2     DREKIKEYNDKIKNRDKKWDSVLYDSNHMRYIYKNASGWRNLEFLK 223
P21CDO-S2_Csep1C      DIEKTKEYNDKMKNGDEKWNSVLYDYQHMRYIYKDATAWKLL--LY 219
H21O-S1_Csep1C        DREKTKEYNDKMKNGDEKWNSVLYDYQHIRYIYKDASAWKLL--IN 220
                      : .* :***:*:*. ::**:**:** :* *:     :.   *
```
