# Supplementary material for: Genomic analysis of oral Campylobacter concisus strains identified a potential bacterial molecular marker associated with active Crohn’s disease
Source: Emerg Microbes Infect. 2018 Apr 11;7:64. doi: 10.1038/s41426-018-0065-6 (PMC5893538; doi:10.1038/s41426-018-0065-6)
Supplement: Supplementary file 8 — Supplementary Figure S6 [file 41426_2018_65_MOESM8_ESM.pdf]

Supplementary Figure S6

A

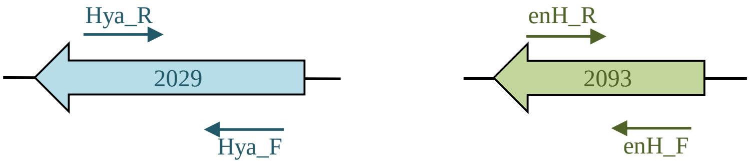

Gene	Locus tag	Primer name	Primer sequence (5'→3')	Annealing temp (°C)	Cycles	Size (bp)
Hypothetical_1	CCS77_2029	Hya_F	ATGACCGCAACATAGGGTGG	60	35	113
		Hya_R	GTCACAACCGACCTCATGGT			
Hypothetical_2	CCS77_2093	enH_F	GGCGTGGAAGTCAAAGAAGA	59		112
		enH_R	TTTCAAGTGTTCCAAGCGAGA			

B

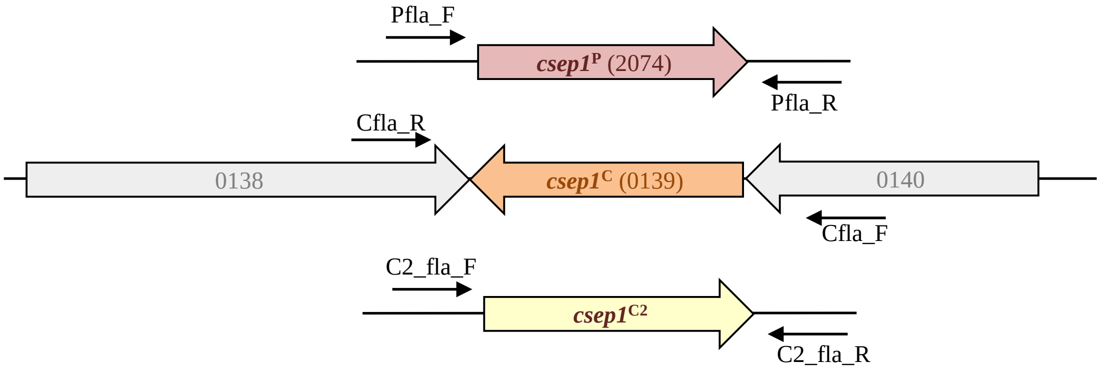

Target	Primer name	Primer sequence (5'→3')	Annealing temp (°C)	Cycles	Size (bp)
Flanking sequences of *csep1*^{P} (CCS77_2074)	Pfla_F	TGCCATGGAGCTTTGGCTTA	59	35	964
	Pfla_R	TGGTCTGGCTATCGTTGGAA			
Flanking sequences of *csep1*^{C} (CCS77_0139)	Cfla_F	CCTGATGTGGTTGAGAGTGT	59		1300-700
	Cfla_R	GGGCAGATCAAGACAAGGCT			
Flanking sequences of *csep1*^{C2}	C2fla_F	GCTTTTTGAAATTATAGCCACTTCC	58		924
	C2fla_R	TTCAAAAATGCACCACTGGAGAA			

C

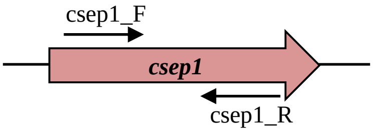

Gene	Primer name	Primer sequence (5'→3')	Annealing temp (°C)	Cycles	Size (bp)
csep1	csep1_F	GCTTCAGTCTTAGCCAGCGT	58	35	454
	csep1_R	CATTGCAGCCATATTCAAGC			
