# Supplementary material for: Genomic analysis of oral Campylobacter concisus strains identified a potential bacterial molecular marker associated with active Crohn’s disease
Source: Emerg Microbes Infect. 2018 Apr 11;7:64. doi: 10.1038/s41426-018-0065-6 (PMC5893538; doi:10.1038/s41426-018-0065-6)
Supplement: Supplementary file 9 — Supplementary Figure legends [file 41426_2018_65_MOESM9_ESM.docx]

**LEGENDS FOR SUPPLEMENTARY FIGURES**

**Supplementary Figure S1 The phylogenetic tree generated based on *C. concisus* 23S rRNA.** The phylogenetic tree based on the 23S rRNA of 63 oral *C. concisus* strains (the genomes of 38 strains were sequenced in this study) was generated to show the genomospecies of these strains. The phylogenetic tree was generated using maximum likelihood method. Strains from active CD, active UC, remission CD and healthy controls were coloured in red, blue, orange and green respectively. The 23S rRNA gene from *Campylobacter jejuni* strain NCTC11168 was used as outgroup. The values on the internal branches correspond to the *bootstrap* values. *Bootstrap* values were generated from 1000 replicates. *Bootstrap* values of more than 70 were indicated. The scale bar is the distance between the strains. GS: genomospecies.

**Supplementary Figure S2 Identification of a novel plasmid in *C. concisus* strains.** **A:** Comparative genomic analysis of 63 oral *C. concisus* strains revealed a genomic fragment that was only present in three strains isolated from two relapsed CD patients with previous ileocecal resection due to small bowel stricture including P2CDO4 (contig 6), P20CDO-S2 (contig 9 and 10), and P20CDO-S3 (contig 9). Strain P2CDO4 was used as the reference strain. **B:** Alignment of the P2CDO4 genome sequenced by MiSeq and PacBio methods showed identical match except that contig 6 from the MiSeq method was missing in contig 2 from the Pacbio method. Genomes were aligned using Mavue. The line above indicates nucleotide position. The local collinear blocks with colours represent the genome fragments that were conserved between genomes. The connecting lines between blocks indicate the location of each rectangle in each genome. Genome sizes between panels A and B were not on scale.

**Supplementary Figure S3** **Sequences of *csep1* genes in *C. concisus* strains isolated from patients with inflammatory bowel disease and healthy controls.** The nucleotide sequences of 26 *csep1* genes were aligned and coloured in red, blue, orange and green accordingly to their phylogenetic groups. Nucleotide insertions at six positions were shaded in grey and labelled as A-F. The six bp insertions at position F (654-659 bp) were only found in strains from active IBD particularly in patients with CD.

**Supplementary Figure S4** **Sequences of** **Csep1 proteins in *C. concisus* strains isolated from patients with inflammatory bowel disease and healthy controls.** The sequences of 26 Csep1 proteins were aligned and coloured in red, blue, orange and green accordingly to their phylogenetic groups. The signal peptides of Csep1 proteins were underlined. Unique amino acids of Csep1 proteins in each group were shaded in grey.

**Supplementary Figure S5 Genomic fragments in *C. concisus* strains AAUH-11UCo and AAUH-11UCdes-a that are similar to the pICON plasmid.** The pICON plasmid from *C. concisus* strain P2CDO4 was compared with the genomes of AAUH-11UCo and AAUH-11UCdes-a strains using BLASTn. The query is the pICON plasmid from *C. concisus* strain P2CDO4. Congits from strains AAUH-11UCo and AAUH-11UCdes-a sharing similarities with the pICON plasmid from strain P2CDO4 were represented by the red lines. Each red line represents one contig.

**Supplementary Figure S6 PCR methods used for detection of pICON plasmid and *csep1* genes. A:** CCS77_2029 and CCS77_2093 were genes exclusively found in pICON plasmid, thus they were used as targets for detection of pICON plasmid in *C. concisus* strains. **B:** For detection of *csep1*^P^, *csep1*^C^, and *csep1*^C2^ genes, primers were designed to target the flanking sequences. **C:** Strains that showed negative results for PCRs targeting the flanking sequences were then subjected to another PCR targeting the conserved regions within the *csep1* genes.
